# Global, Regional, and National Burden of Ectopic Pregnancy: A 30-Year Observational Database Study

**DOI:** 10.1155/2023/3927337

**Published:** 2023-12-18

**Authors:** Wang Bo, Zhang Qianyu, Li Mo

**Affiliations:** ^1^Department of Obstetrics and Gynecology, Tongji Hospital, Tongji Medical College, Huazhong University of Science and Technology, Wuhan, China; ^2^National Clinical Research Center for Obstetrical and Gynecological Diseases, Wuhan, China; ^3^Key Laboratory of Cancer Invasion and Metastasis, Ministry of Education, Wuhan, China

## Abstract

**Objective:**

To estimate global, regional, and national trends due to ectopic pregnancy as part of the 2019 Global Burden of Disease study.

**Methods:**

We systematically reviewed trends in ectopic pregnancy burden using data from the Global Burden of Disease (GBD) database, including 21 regions, 195 countries, and territories over the past 30 years. The trends of ectopic pregnancy-related incidence, mortality, and disability-adjusted life years (DALYs) attributable to all known risk factors were also analyzed. Age-standardized rates (ASRs) and their estimated annual percentage changes (EAPCs) were also calculated.

**Results:**

Incident cases, deaths, and DALYs of ectopic pregnancy increased worldwide in the past 30 years. The age-standardized incidence rate (ASIR) was decreasing (EAPC = −1.14, 95% confidence interval (CI): −1.29 to −0.98), and the age-standardized death (EAPC = −0.9, 95% CI: −1.03 to −0.76) and DALY rate decreased generally (EAPC = −0.83, 95% CI: −0.98 to −0.68). In addition, the burden of ectopic pregnancy is lower in areas with higher socioeconomic development, and significant positive correlations between ASRs and sociodemographic index (SDI) were observed, especially among low-middle SDI, and low SDI quintiles carried the majority burden of ectopic pregnancy.

**Conclusion:**

Globally, the incidence, mortality, and DALY rate of ectopic pregnancy had been decreasing from 1990 to 2019. Compared with lower and decreasing ASIR in the high SDI region, ASIR in the low SDI region was always high, indicating the need for ectopic pregnancy treatment improvement and the establishment of more targeted and specific strategies in low SDI countries to reduce the incidence of ectopic pregnancy.

## 1. Introduction

Worldwide, ectopic pregnancy, as one of the common acute disease occurring at abdomen in obstetrics and gynecology, is the main cause of maternal death [1, 2]. Once the ectopic pregnancy site ruptures, it may cause serious complications such as bleeding, or even shock, which may endanger life [3, 4]. Nowadays, with the development of the social economy and the change in people's lifestyles, the incidence rate and clinical treatment of ectopic pregnancy have also changed greatly, and the mortality of ectopic pregnancy is also decreasing [5, 6]. Of these, actively tracking changes in the burden of ectopic pregnancy is quite necessary, which can provide relevant data support for better implementation of prevention and treatment, especially for the improvement of health decision-making and disease management in various countries and regions.

Previous studies mainly focused on regional studies, and most of them were single-center studies [7–10]. Limited by population sample size and time span, it was difficult to accurately trace the incidence trend of ectopic pregnancy, so more comprehensive and extensive data were urgently needed as support. Nowadays, with the increasing improvement of public databases, making full use of data mining technology can search for potentially valuable knowledge from a large amount of data, thereby better serving clinical applications [11, 12]. Fortunately, the global burden of disease meets the needs of time and space span analysis of ectopic pregnancy, especially from the relevant robust indicators of epidemiology (such as age-standardized incidence rate (ASIR) and age-standardized death rate (ASDR)), which can more accurately assess the incidence of ectopic pregnancy and its prevention and treatment effects, thus providing an important basis for the formulation of future public health strategies related to ectopic pregnancy [13, 14]. Given this situation, this study reviewed the progress related to the epidemiology of ectopic pregnancy in the past 30 years in order to provide a theoretical basis for clinical workers to treat and care for ectopic pregnancy.

## 2. Materials and Methods

### 2.1. Data Resources

To obtain detailed information on the global disease burden database of ectopic pregnancy, we obtained it with the help of the Global Health Data Exchange (GHDx) query tool (download address: https://ghdx.healthdata.org/gbd-results-tool). According to the sociodemographic index (SDI), regions and countries were divided into five levels: low SDI, low-middle SDI, middle SDI, high-middle SDI, and high SDI. In addition, according to geographical characteristics, there were twenty-one geographical regions including East Asia, South Asia, and Western Europe.

### 2.2. Evaluation Index for Ectopic Pregnancy

In short, from this database, we can obtain detailed data on the number of cases and deaths in the world, countries, and regions in the current year and calculate the relevant “age-standardized rate (ASR)” and its estimated annual percentage changes (EAPCs) through the number of cases and deaths of ectopic pregnancy from 1990 to 2019.

(1) $$\begin{array}{c} \text{ASR}=\frac{\sum_{i=1}^{A}a1wi}{\sum_{i=1}^{A}wi}\times 100,000. \end{array}$$

In this formula, *a* represents the specific age rate of the *i*th age group, *wi* represents the population (or weight) of the corresponding *i*th age subgroup in the selected reference standard population, and *A* represents the number of age groups. These indicators can better evaluate the changing trend related to the incidence and death of ectopic pregnancy in the past 30 years. In addition, disability-adjusted life years (DALYs) refer to all the years of healthy life lost from illness to death, including years of life lost due to early death and years of healthy life lost due to disability. In this study, the DALYs were the sum of the years of lost life (YLL) and the years lived with disability (YLD).

### 2.3. Statistical Methods

In this study, the EAPC calculation formula was 100 × [exp(*β*) − 1], and 95% confidence interval (CI) was calculated with a linear regression model. When the EAPC value and its 95% CI > 0, it was defined as a trend increase. On the contrary, when the EAPC value and its 95% CI < 0, it indicated that the trend decreases. Others represent that ASR was relatively stable in a period. To explore the influencing factors of EAPC, the Pearson correlation analysis in R software (download address: https://www.r-project.org/) was used to evaluate the correlation between EAPCs and ASR in 1990 and the correlation between EAPCs and Human Development Index (HDI) in 2019, as well as to draw a Choropleth chart.

## 3. Results

### 3.1. The Change in the Incidence of Ectopic Pregnancy

**Figure 1:**
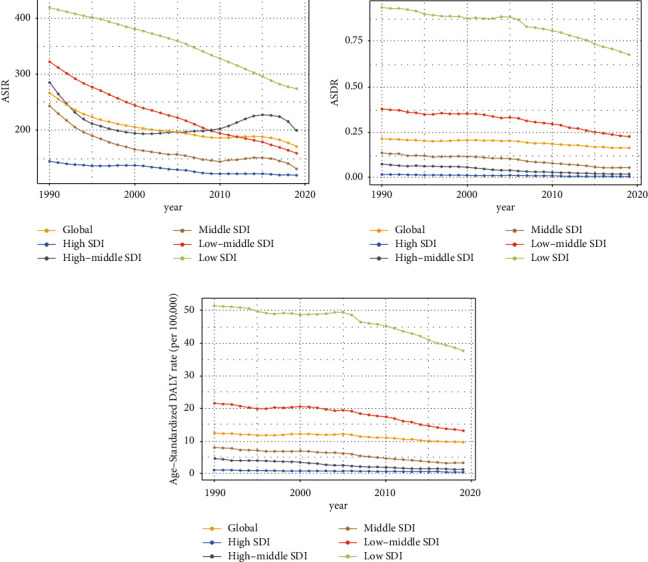
The trends of age-standardized incidence (a), death (b), and DALY (c) rate from 1990 to 2019 among different SDI quintiles.

**Figure 2:**
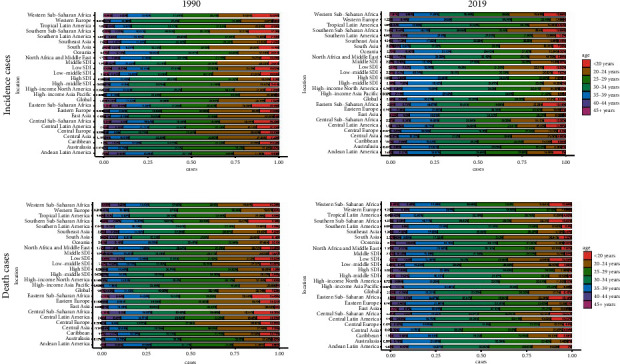
The composition of ectopic pregnancy incidence/death of different ages by region. (a) Incidence in 1990. (b) Incidence in 2019. (c) Death rate in 2019. (d) Death rate in 2019.

**Figure 3:**
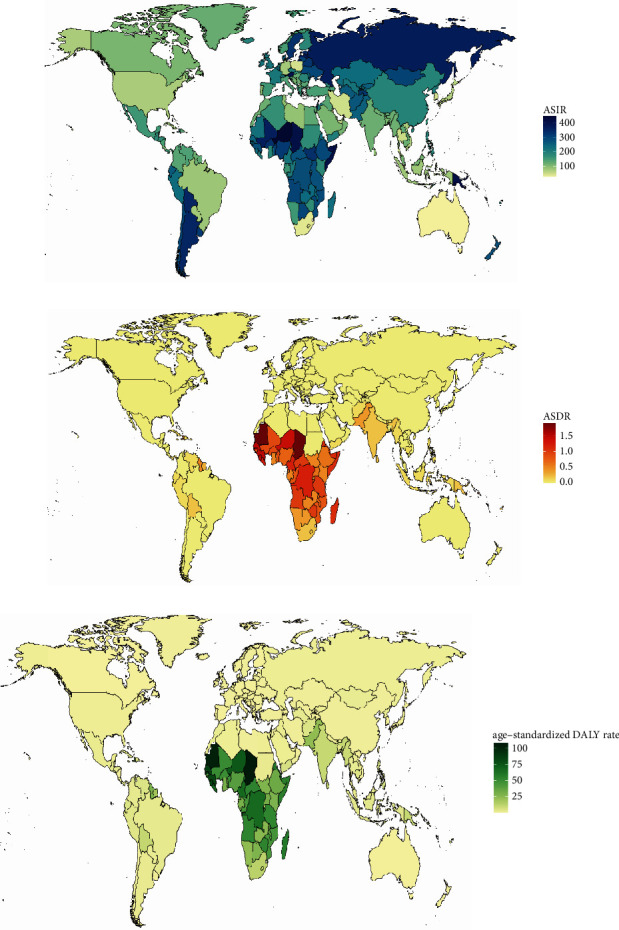
The worldwide ASRs (per 100,000 population) of ectopic pregnancy incidence, death, and DALY in 194 countries in 2019. (a) ASIR. (b) ASDR. (c) Age-standardized DALY rate. ASIR: age-standardized incidence rate; ASDR: age-standardized death rate; and DALY: disability-adjusted life year.

**Figure 4:**
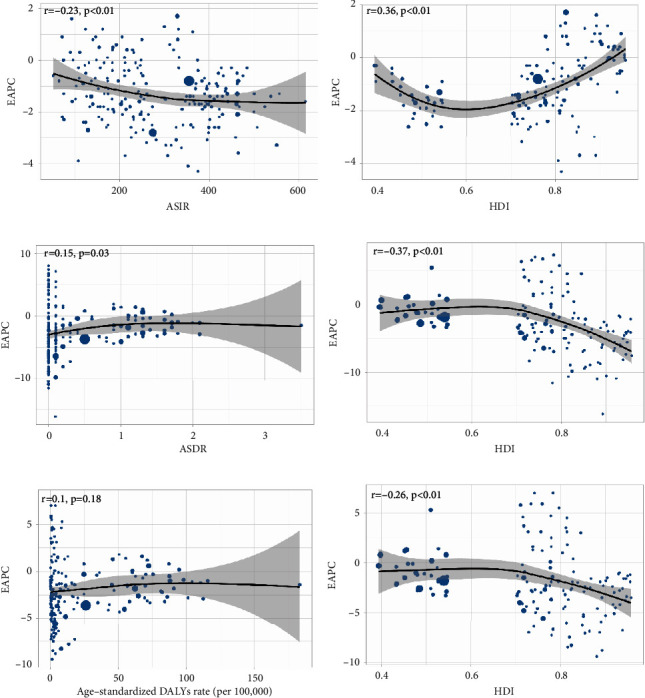
The correlations between EAPC and age-standardized incidence (ASIR) (a), death (ASDR) (c), and DALY (e) rate in 1990 and correlations between EAPC of incidence (b), death (d), DALY (f), and HDI in 2019. Each circle represents a country and the size represents number of ectopic pregnancy patients. The *r* value is the correlation coefficient of Pearson's correlation. EAPC: estimated annual percentage change and HDI: human development index.

In the past 30 years, the annual incidence decreased gradually and there were 66924.05 (95% UI, 52254.01∼85985.7) × 10^2^ incidences in 2019 and 74532.67 (95% UI, 57389.85∼95570.78) × 10^2^ incidents in 1990 at the global level (Table 1 and Figure 1). Contrary to the 10.21% decrease (EAPC = −1.14, 95 CI%: −1.29∼−0.98) in incidences over the past 30 years, the ASIR was stable with 131.91/100,000 persons (95% UI, 101.02∼169.66) in 1990 to 84.33/100,000 persons (95% UI, 65.91∼108.27) in 2019 (Figure 2). In addition, from the perspective of different SDI divisions, the declining trend of EAPC in high SDI (EAPC = −0.74, 95 CI%: −0.8∼−0.67) regions was generally lower than that in low SDI (EAPC = −1.46, 95 CI%: −1.55∼−1.37) and low-middle SDI regions (EAPC = −2.25, 95 CI%: −2.29∼−2.21) (Supplementary Figures 1 and 2). Consistent with the results, the three countries with the most significant decline in EAPC were Qatar, Afghanistan, and Somalia. The three countries with the lowest decline in EAPC were the Northern Mariana Islands, Albania, and Puerto Rico (Figure 3, Supplementary Tables 1 and 3). However, from the regional perspective, the decline of EAPC in East Asia, high-income North America, and Central Europe was the slowest, while the declining trend of EAPC in Oceania, Western Sub-Saharan Africa, and Central Sub-Saharan Africa ranked in the top three (Supplementary Table 2 and Supplementary Figure 3). In addition, we also found a negative correlation between EAPC and ASIR (*ρ* = −0.23 and *p* < 0.01) and a positive correlation between EAPC and the SDI (*ρ* = 0.36 and *p* < 0.01) (Figure 4). Collectively, the incidence of ectopic pregnancy was quite different in various countries and regions, and the overall trend of incidence rate in developed countries was lower than that in less developed countries, although the decline of EAPC in the past three decades was less than that in less developed countries and regions.

### 3.2. Trends in Ectopic Pregnancy-Related Deaths

At the global level, ectopic pregnancy-related deaths cannot be ignored. As shown in Table 2 and Figure 1, the annual deaths decreased gradually and there were 64.52 (95% UI, 54.96∼75.13) × 10^2^ deaths in 2019 and 57.49 (95% UI, 51.07∼64.35) × 10^2^ deaths in 1990. In the past 30 years, the ASDR was stable with 0.11/100,000 persons (95% UI, 0.09∼0.12) in 1990 to 0.08/100,000 persons (95% UI, 0.07∼0.09) in 2019. Fortunately, the EAPC of ectopic pregnancy still maintained a weak downward trend, which indicated that the mortality rate was effectively controlled. In addition, from the perspective of different SDI regions, the decline of EAPC in high SDI (EAPC = −3.02, 95% CI: −3.2∼−2.83) and high-middle SDI (EAPC = −4.98, 95% CI: −5.25∼−4.71) regions was significantly greater than that in low SDI (EAPC = −1.00, 95% CI: −1.13∼−0.88) regions; especially the ASDR of regions above high-middle SDI in 2019 has been significantly lower than 10/100000 (Figure 2 and Supplementary Figures 1 and 2). From the national and regional level, as shown in Figure 3 and Supplementary Table 1, the three countries with the highest and lowest decline in EAPC were Ecuador, Belize, Jamaica, Estonia, Czechia, and Hungary. However, the three regions with the largest and lowest decline in EAPC were the Caribbean, Andean Latin America, Central Sub-Saharan Africa, Eastern Europe, Central Europe, and East Asia (Supplementary Tables 2 and 4 and Supplementary Figure 3). In addition, we also found a positive correlation between EAPC and ASDR (*ρ* = 0.15, *p* < 0.05) and a negative correlation between EAPC and the SDI (*ρ* = −0.37, *p* < 0.01) (Figure 4). In general, the mortality due to ectopic pregnancy in both developed and underdeveloped areas was on the decline, which also reflected the global efforts to control ectopic pregnancy-related deaths.

### 3.3. The Change in DALYs of Ectopic Pregnancy

At the global level, there were 3394.42 (95% UI, 3017.69∼3786.49) × 10^2^ DALYs in 1990 and 3780.28 (95% UI, 3225.46∼4400.9) × 10^2^ DALYs in 2019. The number of DALYs was stable over the past 30 years, and the age-standardized DALY rate decreased significantly with an EAPC of −0.83 (95% CI, from −0.98 to −0.68), dropping from 6.15/100, 000 (95% UI, 5.47–6.86) in 1990 to 4.79/100,000 persons (95% UI, 4.09∼5.59) in 2019 (Table 3 and Figure 1). On analysis from the SDI level, we found that the age-standardized DALY rate in all the SDI regions declined, and the decline of EAPC with high-middle SDI increased, followed by middle SDI (Supplementary Figures 1 and 2). On observation from the GBD regions and countries level, the three countries with the highest age-standardized DALY rate of EAPC were Belize, Ecuador, and Haiti; the three countries with the lowest age-standardized DALY rate of EAPC were Poland, Hungary, and Estonia (Figures 2 and 3 and Supplementary Table 1). In terms of regions, the three regions with the largest and lowest number of DALY were the Caribbean, Andean Latin America, Central Sub-Saharan Africa, Eastern Europe, Central Europe, and East Asia (Supplementary Tables 2 and 5 and Supplementary Figure 3). In addition, we also found a nonsignificant correlation between EAPC and ASDR (*ρ* = 0.1, *p* = 0.18) and a negative correlation between EAPC and the SDI (*ρ* = −0.26, *p* < 0.01) (Figure 4). In summary, the declining trend of DALYs in various countries and regions around the world reflected that the loss of ectopic pregnancy on women's healthy life span was gradually narrowing.

### 3.4. Distribution of Related Risk Factors among SDI Quintiles

**Figure 5:**
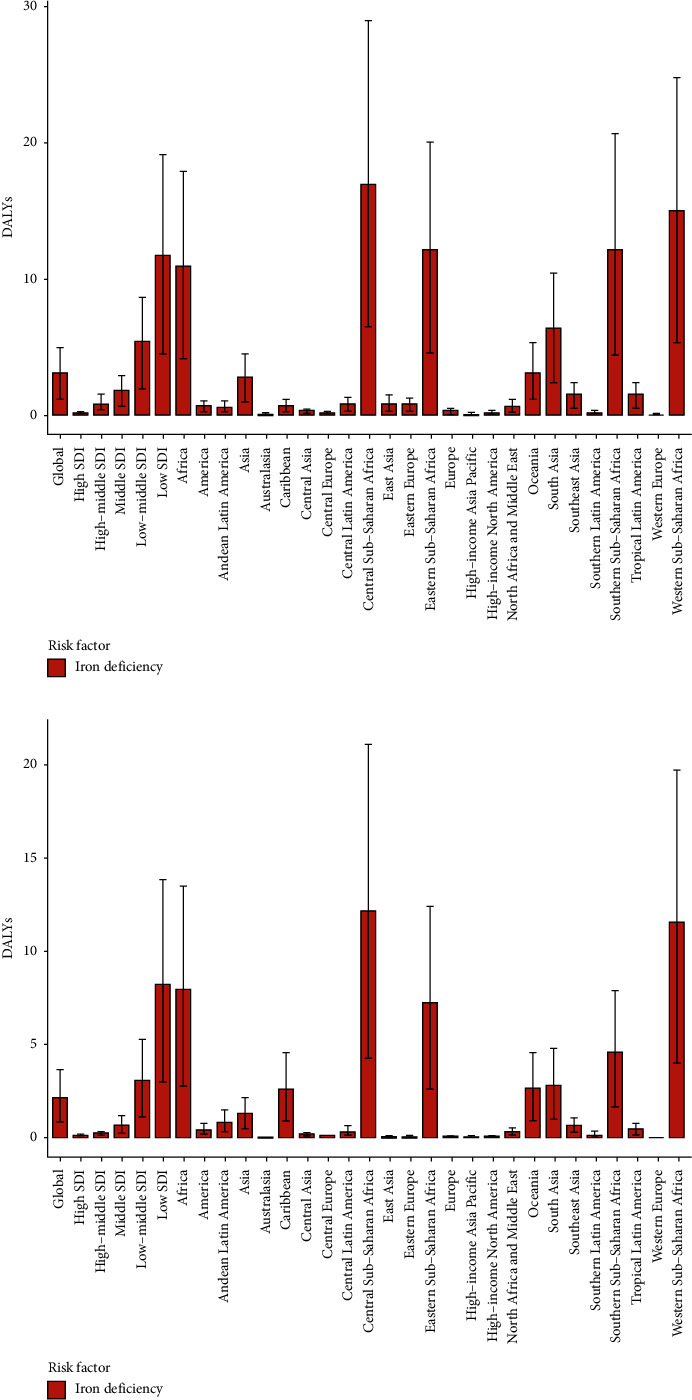
The ectopic pregnancy DALYs attributed to iron deficiency in 1990 (a) and 2019 (b). DALY: disability-adjusted life year.

**Figure 6:**
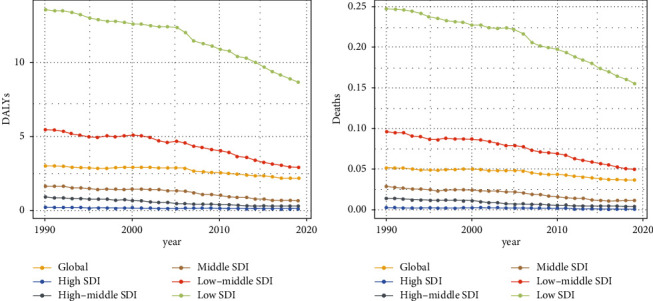
The trends of age-standardized DALYs (a) and deaths (b) attributable to iron deficiency from 1990 to 2019.

According to the potential threat factors provided by GBD, we conducted an attributable risk factor analysis, which showed that iron deficiency was significantly associated with ectopic pregnancy-related deaths and DALYs. As shown in Figure 5, compared with 1990, the risk factors of iron deficiency in a few regions in 2019, such as South Asia and Western Europe, have been significantly reduced. Meanwhile, from the perspective of different SDI regions, iron deficiency in low-grade SDI regions was the most significant, followed by middle and low-middle SDI regions, while the risk factors for iron deficiency in the middle, high-middle, and high SDI regions were relatively stable (Figure 6). However, the factors of death and DALYs caused by iron deficiency in the overall SDI region in the past 30 years also showed a downward trend, even in underdeveloped regions.

## 4. Discussion

This study systematically elaborated the latest trends and models of global incidence rate, mortality, and DALY of ectopic pregnancy from 1990 to 2019, based on the results of GBD 2019. Compared with the previous research reports, we have made a comprehensive summary based on the incidence trend of ectopic pregnancy in various countries and regions around the world, and we firmly believe that this has a very cutting-edge guiding significance for the timely follow-up of the prevention and control of ectopic pregnancy.

Although ectopic pregnancy is a benign gynecological disease, its sudden onset may bring high-risk factors such as hemorrhagic shock, which will pose a potential threat to the lives of patients. Worldwide, the early identification and management of ectopic pregnancy still show great differences, which are closely related to the health policies of various countries and regions. For example, the total proportion of ectopic pregnancy reported in North America and other regions is 19.7/1000, which is the main cause of death for women in early pregnancy, accounting for 10% to 15% of all women's deaths [1]. In the past 30 years, for every 1000 pregnancies in Northern Europe, ectopic pregnancy has increased from 11.2 to 18.8. In 1970, 17800 cases of ectopic pregnancy were hospitalized in the United States, which rose to 88400 in 1989. More than 10000 ectopic pregnancies are admitted to hospital every year in the UK (the incidence rate is about 11.5/1000 pregnancies) [15, 16]. The burden of the disease varies greatly across the globe, and this imbalance is mainly affected by population structure and socioeconomic status [17, 18].

In this study, the number of morbidity and ectopic pregnancy-related deaths in developed regions is significantly lower than that in underdeveloped regions. As for the change range of ASIR and ASDR, even though the decline in developed regions is not significant, compared with the overall population base, the prevention, and control of the fatal risk of ectopic pregnancy tends to be more stable, which also suggests that early management strategies for ectopic pregnancy, especially in underdeveloped regions. Because of the existing limitations of medical resources and health systems, the burden of ectopic pregnancy remains an important challenge in the world and a few underdeveloped regions.

Ectopic pregnancy is still a clear public health burden and a public health issue [19]. So far, people have insufficient knowledge of ectopic pregnancy, especially in underdeveloped countries and regions, and there is often greater neglect of the risk factors of ectopic pregnancy. Previous studies have demonstrated that the occurrence of ectopic pregnancy is the result of a combination of one or more factors, which may be related to reproductive system infection, abortion, fallopian tube surgery, history of ectopic pregnancy, the use of intrauterine devices, cesarean section, emergency contraception, and other factors [1, 20–22]. Many unknown potential risk factors are still worth discussing. For example, this study found that iron deficiency may become a high-risk factor for ectopic pregnancy, especially in regional distribution.

Previous studies have shown that menstruation loss and a significant increase in iron demand during pregnancy are the main reasons for negative iron balance in women of childbearing age and pregnant women [23, 24]. For example, the World Health Organization recommends that pregnant women take 300 micrograms of iron and 400 micrograms of folic acid daily. Studies on iron supplementation showed that the risk of anemia in pregnant women at term was reduced by 70%, and the risk of iron deficiency at term was reduced by 57%. At the same time, the level of hemoglobin per liter of women receiving iron therapy at term or in the near future was significantly higher than that of women not receiving supplements [25]. Therefore, the risk of iron deficiency during pregnancy and lactation mainly stems from insufficient iron reserves in women of childbearing age before pregnancy. We speculate that this may also lead to the abnormal development of fertilized eggs, which increases the risk of ectopic pregnancy.

Our research also inevitably has the following limitations. First, there was a lack of data for countries with low socioeconomic status and the least data sources, which may offset the assessment of the disease burden of these countries and regions. In the future, it is still necessary to strengthen the data-sharing policy to expand the accurate assessment of the burden of ectopic pregnancy in all regions of the world. Second, because the GBD database did not report the disease burden of ectopic pregnancy by type, it is necessary to carefully evaluate the pregnancy risk of different ectopic parts in the future, especially the ectopic pregnancy in the ampulla and interstitial parts. We believe that the disease burden of ectopic pregnancy in different parts is also different. Third, the lack of data on some other potential risk factors of ectopic pregnancy may hinder the interpretation of this association. Therefore, it is still necessary to expand the screening of risk factors of ectopic pregnancy, and the risk factors in different regions depend on each other, so it is necessary to treat with caution and formulate corresponding policies. Notwithstanding, our study provides an updated estimate of the burden of ectopic pregnancy and its causes, which will be useful for public health policymakers.

## 5. Conclusion

In summary, ectopic pregnancy is a major public health problem worldwide, but there are great differences among countries and regions. Although the prevalence rate of age-standardized point and DALY rate caused by ectopic pregnancy has decreased in the past 30 years, the burden is still heavy, especially in less-developed countries. In addition, despite the multifactorial nature of the disease, dietary iron defense is still an important factor that cannot be ignored in all regions of ectopic pregnancy. Therefore, it is necessary to pay more attention to nutritional interventions, formulate comprehensive interventions, give priority to the highest-risk group, and strengthen the early prevention and treatment of ectopic pregnancy to improve the prognosis of patients.

## Figures and Tables

**Table 1:** The incident cases and ASIR in 1990 and 2019 and its temporal trends.

	1990		2019		1990–2019
	Incident cases no. 10^2^ (95% UI)	ASIR per 100,000 no. (95% UI)	Incident cases no. 10^2^ (95% UI)	ASIR per 100,000 no. (95% UI)	EAPC no. (95% CI)
Global	74532.67 (57389.85∼95570.78)	131.91 (101.02∼169.66)	66924.05 (52254.01∼85985.7)	84.33 (65.91∼108.27)	−1.14 (−1.29∼−0.98)
Sociodemographic index					
High SDI	6242.86 (4667.36∼8338.58)	71.17 (53.12∼94.37)	5531.62 (4348.36∼7049.7)	57.62 (45.73∼74.1)	−0.74 (−0.8∼−0.67)
High-middle SDI	17951.77 (13620.79∼23412.41)	140.29 (106.5∼183.08)	14617.31 (11210.83∼19318.07)	96.51 (74.53∼125.84)	−0.36 (−0.73∼0.02)
Middle SDI	22689.55 (17171.55∼29557.96)	119.8 (90.21∼157.33)	16593.44 (12670.09∼21895.64)	64.94 (49.78∼85.01)	−1.54 (−1.8∼−1.29)
Low-middle SDI	17784.67 (13690.53∼22813.84)	159.35 (123.31∼204.5)	15124.39 (11603.3∼19769.01)	79.17 (61∼103.5)	−2.25 (−2.29∼−2.21)
Low SDI	9831.77 (7654.99∼12927.01)	211.4 (164.5∼276.63)	15016.75 (11562.32∼19829.5)	138.54 (106.83∼182.02)	−1.46 (−1.55∼−1.37)
Region					
Andean Latin America	759.22 (584.87∼985.74)	206.5 (158.5∼272.5)	895.2 (681.3∼1172.7)	133.4 (101.8∼174.8)	−1.3 (−1.5∼−1.2)
Australasia	90.11 (63.09∼132.1)	41.3 (28.9∼60)	91.4 (71.5∼117.3)	33.2 (26.1∼42.8)	−0.6 (−0.9∼−0.2)
Caribbean	343.62 (259.49∼456.28)	91.9 (69.1∼122.2)	340.9 (256.2∼457.5)	70.9 (53.4∼95.4)	−0.8 (−0.9∼−0.7)
Central Asia	1184.62 (880.21∼1565.94)	161.4 (120.9∼212.2)	1171.3 (867.7∼1548.6)	114.1 (84.8∼150.2)	−0.7 (−1.1∼−0.4)
Central Europe	818.49 (618.01∼1073.24)	70.5 (52.8∼92.9)	576.9 (443.9∼744.9)	57.4 (43.8∼74)	−0.4 (−0.7∼0)
Central Latin America	1958.09 (1455.52∼2646.62)	119.6 (88.7∼161.7)	2071 (1617.5∼2705.1)	78.2 (60.9∼102.2)	−1.9 (−2.1∼−1.7)
Central Sub-Saharan Africa	1055.47 (809.63∼1415.48)	219.7 (166.7∼291.8)	1756.1 (1320.5∼2376.7)	142.2 (106.4∼193)	−1.3 (−1.5∼−1.2)
East Asia	25570.3 (18989.16∼33700.89)	172.9 (128∼229.3)	14629.6 (11083.1∼19701)	93.9 (71.3∼124.4)	−0.8 (−1.3∼−0.3)
Eastern Europe	3607.84 (2732.09∼4770.29)	163.6 (124.8∼217.2)	3501.8 (2609.2∼4695.8)	173.8 (131∼227.1)	1.5 (0.9∼2)
Eastern Sub-Saharan Africa	3591.3 (2763.85∼4735.98)	219.3 (168.7∼289.8)	5504.6 (4216.6∼7357.4)	138.9 (106∼185.2)	−1.6 (−1.7∼−1.5)
High-income Asia Pacific	642.48 (461.03∼879.71)	36.8 (26.3∼50.6)	460 (353.3∼605.7)	29.8 (23∼39.5)	−0.8 (−1∼−0.6)
High-income North America	1946.98 (1372.99∼2689.69)	63.3 (44.8∼87.3)	1325.3 (1111.3∼1628.9)	39.7 (33.3∼48.8)	−2.4 (−2.8∼−1.9)
North Africa and Middle East	3890.95 (3002.2∼5107.44)	119.8 (92.6∼157.8)	4433.7 (3382.3∼5950.9)	64.2 (49.1∼85.8)	−2.1 (−2.2∼−2)
Oceania	129.7 (96.9∼173.72)	209 (156.6∼277.1)	253.7 (190.1∼337)	187.2 (139.4∼249.5)	−0.4 (−0.4∼−0.4)
South Asia	15625.16 (11928.94∼20727.44)	141.4 (108.4∼187.2)	13519.4 (9924∼18087.5)	67 (49.7∼89.5)	−2.6 (−2.7∼−2.6)
Southeast Asia	4043.74 (3077.02∼5344.98)	83.9 (64.1∼111.4)	3777.9 (2889.8∼5068)	52 (39.6∼69.5)	−1.5 (−1.6∼−1.5)
Southern Latin America	1123.21 (844.51∼1483.53)	226.9 (170.6∼299)	1169.6 (869.9∼1582)	169.8 (126.5∼227.8)	−0.8 (−0.9∼−0.7)
Southern Sub-Saharan Africa	353.35 (273.27∼458.24)	66 (51.1∼85.6)	359.8 (277.5∼469.2)	40.8 (31.6∼53.1)	−1.6 (−1.7∼−1.5)
Tropical Latin America	991.05 (761.72∼1296.88)	60.5 (46.6∼79.2)	1035.1 (777.4∼1405.1)	43.1 (32.4∼58.2)	−1 (−1.1∼−0.9)
Western Europe	3115.61 (2332.46∼4152.94)	79.1 (59.2∼105.2)	3413 (2591.1∼4440.7)	89.4 (68∼117.5)	0.7 (0.6∼0.8)
Western Sub-Saharan Africa	3691.38 (2862.65∼4859.65)	217.5 (168.3∼286.7)	6637.7 (5095.3∼8847.2)	156.9 (119.9∼209)	−1.1 (−1.2∼−1)

ASIR: age-standardized incidence rate.

**Table 2:** The death cases and ASDR in 1990 and 2019 and its temporal trends.

	1990		2019		1990–2019
	Death cases no. 10^2^ (95% UI)	ASDR per 100,000 no. (95% UI)	Death cases no. 10^2^ (95% UI)	ASDR per 100,000 no. (95% UI)	EAPC no. (95% CI)
Global	57.49 (51.07∼64.35)	0.11 (0.09∼0.12)	64.52 (54.96∼75.13)	0.08 (0.07∼0.09)	−0.9 (−1.03∼−0.76)
Sociodemographic index					
High SDI	0.78 (0.7∼0.87)	0.01 (0.01∼0.01)	0.32 (0.28∼0.36)	0 (0∼0)	−3.02 (−3.2∼−2.83)
High-middle SDI	4.6 (4.12∼5.14)	0.04 (0.03∼0.04)	1.34 (1.15∼1.55)	0.01 (0.01∼0.01)	−4.98 (−5.25∼−4.71)
Middle SDI	12.09 (10.75∼13.45)	0.07 (0.06∼0.07)	6.79 (5.75∼8.02)	0.03 (0.02∼0.03)	−3.25 (−3.59∼−2.91)
Low-middle SDI	19.68 (16.98∼22.79)	0.19 (0.16∼0.22)	21.26 (17.78∼24.95)	0.11 (0.09∼0.13)	−1.63 (−1.83∼−1.43)
Low SDI	20.32 (17.23∼23.59)	0.47 (0.4∼0.55)	34.78 (28.49∼41.87)	0.34 (0.28∼0.41)	−1 (−1.13∼−0.88)
Region					
Andean Latin America	0.08 (0.06∼0.09)	0.02 (0.02∼0.02)	0.28 (0.2∼0.37)	0.04 (0.03∼0.06)	2.26 (1.17∼3.36)
Australasia	0.01 (0.01∼0.02)	0.01 (0∼0.01)	0.01 (0∼0.01)	0 (0∼0)	−3.87 (−4.68∼−3.07)
Caribbean	0.09 (0.07∼0.11)	0.02 (0.02∼0.03)	0.51 (0.37∼0.67)	0.11 (0.08∼0.14)	6.29 (5.36∼7.22)
Central Asia	0.06 (0.06∼0.07)	0.01 (0.01∼0.01)	0.04 (0.04∼0.05)	0 (0∼0.01)	−2.44 (−2.58∼−2.3)
Central Europe	0.12 (0.11∼0.14)	0.01 (0.01∼0.01)	0.01 (0.01∼0.01)	0 (0∼0)	−7.85 (−8.37∼−7.32)
Central Latin America	0.69 (0.62∼0.76)	0.04 (0.04∼0.05)	0.51 (0.42∼0.64)	0.02 (0.02∼0.02)	−2.65 (−2.99∼−2.32)
Central Sub-Saharan Africa	2.89 (2.17∼3.69)	0.64 (0.48∼0.83)	6.43 (4.68∼8.16)	0.55 (0.4∼0.72)	0.23 (−0.08∼0.54)
East Asia	4.14 (3.32∼5.12)	0.03 (0.02∼0.04)	0.65 (0.49∼0.82)	0 (0∼0.01)	−6.4 (−6.92∼−5.88)
Eastern Europe	1.12 (0.96∼1.3)	0.05 (0.04∼0.06)	0.08 (0.06∼0.11)	0 (0∼0.01)	−9.35 (−9.79∼−8.91)
Eastern Sub-Saharan Africa	8.48 (7.04∼10)	0.61 (0.5∼0.72)	12.97 (10.44∼15.79)	0.37 (0.3∼0.45)	−1.57 (−1.67∼−1.47)
High-income Asia Pacific	0.1 (0.09∼0.12)	0.01 (0∼0.01)	0.02 (0.01∼0.02)	0 (0∼0)	−5.73 (−5.94∼−5.51)
High-income North America	0.42 (0.35∼0.5)	0.01 (0.01∼0.02)	0.2 (0.16∼0.24)	0.01 (0∼0.01)	−2.33 (−2.65∼−2)
North Africa and Middle East	0.92 (0.8∼1.03)	0.03 (0.03∼0.03)	0.71 (0.57∼0.89)	0.01 (0.01∼0.01)	−3.52 (−3.6∼−3.43)
Oceania	0.07 (0.06∼0.09)	0.11 (0.09∼0.14)	0.13 (0.1∼0.18)	0.1 (0.07∼0.13)	−0.56 (−0.85∼−0.27)
South Asia	20.4 (16.73∼24.71)	0.19 (0.16∼0.24)	17.78 (13.97∼22.12)	0.09 (0.07∼0.11)	−2.88 (−3.15∼−2.6)
Southeast Asia	3.23 (2.73∼3.86)	0.07 (0.06∼0.08)	2.34 (1.92∼2.76)	0.03 (0.03∼0.04)	−2.9 (−3.17∼−2.62)
Southern Latin America	0.05 (0.04∼0.06)	0.01 (0.01∼0.01)	0.07 (0.05∼0.08)	0.01 (0.01∼0.01)	−0.06 (−0.5∼0.38)
Southern Sub-Saharan Africa	2.62 (2.22∼3.08)	0.52 (0.44∼0.6)	1.68 (1.25∼2.17)	0.19 (0.14∼0.25)	−2.08 (−3.17∼−0.98)
Tropical Latin America	0.95 (0.8∼1.11)	0.06 (0.05∼0.07)	0.46 (0.39∼0.54)	0.02 (0.02∼0.02)	−2.82 (−3.57∼−2.06)
Western Europe	0.19 (0.18∼0.21)	0 (0∼0.01)	0.04 (0.04∼0.05)	0 (0∼0)	−5.24 (−5.56∼−4.92)
Western Sub-Saharan Africa	10.86 (8.69∼13.73)	0.67 (0.54∼0.85)	19.59 (15.11∼25.54)	0.48 (0.37∼0.63)	−1.16 (−1.41∼−0.9)

ASDR: age-standardized death rate.

**Table 3:** The DALY and age-standardized DALY rate in 1990 and 2019 and its temporal trends.

	1990		2019		1990–2019
	DALY no. 10^2^ (95% UI)	Age-standardized DALY rate per 100,000 no. (95% UI)	DALY no. 10^2^ (95% UI)	Age-standardized DALY rate per 100,000 no. (95% UI)	EAPC no. (95% CI)
Global	3394.42 (3017.69∼3786.49)	6.15 (5.47∼6.86)	3780.28 (3225.46∼4400.9)	4.79 (4.09∼5.59)	−0.83 (−0.98∼−0.68)
Sociodemographic index					
High SDI	51.23 (45.52∼57.46)	0.59 (0.52∼0.66)	23.33 (19.94∼27.31)	0.25 (0.21∼0.29)	−2.69 (−2.85∼−2.53)
High-middle SDI	287.77 (255.84∼322.42)	2.28 (2.03∼2.55)	90.98 (78.1∼106.25)	0.64 (0.55∼0.75)	−4.58 (−4.81∼−4.35)
Middle SDI	734.25 (653.68∼815.8)	3.96 (3.52∼4.4)	411.3 (350.06∼484.31)	1.64 (1.4∼1.93)	−3.14 (−3.48∼−2.8)
Low-middle SDI	1161.63 (999.62∼1340.97)	10.64 (9.19∼12.3)	1249.17 (1048.39∼1462.47)	6.59 (5.54∼7.72)	−1.56 (−1.79∼−1.33)
Low SDI	1158.45 (982.76∼1348.75)	25.93 (22.03∼30.09)	2003.28 (1642.86∼2399.11)	19.03 (15.57∼22.89)	−0.95 (−1.09∼−0.81)
Region					
Andean Latin America	5.38 (4.5∼6.56)	1.4 (1.17∼1.69)	17.35 (12.9∼22.74)	2.58 (1.92∼3.38)	2 (0.99∼3.02)
Australasia	0.82 (0.66∼1)	0.38 (0.3∼0.46)	0.38 (0.3∼0.47)	0.14 (0.11∼0.17)	−3.34 (−4.02∼−2.65)
Caribbean	5.46 (4.54∼6.68)	1.45 (1.2∼1.77)	29.74 (22.15∼38.84)	6.18 (4.6∼8.06)	6.07 (5.17∼6.97)
Central Asia	4.76 (4.08∼5.6)	0.66 (0.57∼0.77)	3.67 (2.95∼4.5)	0.36 (0.29∼0.44)	−1.97 (−2.14∼−1.79)
Central Europe	7.76 (7.06∼8.58)	0.64 (0.58∼0.71)	1.24 (0.96∼1.58)	0.12 (0.1∼0.16)	−5.91 (−6.48∼−5.33)
Central Latin America	43.33 (38.93∼48.31)	2.53 (2.28∼2.82)	32.71 (26.9∼40.27)	1.23 (1.01∼1.51)	−2.56 (−2.88∼−2.24)
Central Sub-Saharan Africa	166 (125.91∼210.78)	35.59 (26.75∼45.43)	367.59 (267.02∼461.86)	30.58 (22.4∼38.92)	0.24 (−0.07∼0.56)
East Asia	272.15 (221.61∼331.39)	1.86 (1.51∼2.27)	50.8 (39.85∼62.69)	0.34 (0.27∼0.42)	−5.68 (−6.12∼−5.24)
Eastern Europe	68.18 (58.15∼79.1)	2.94 (2.5∼3.42)	8.06 (6.07∼10.5)	0.4 (0.31∼0.52)	−7.65 (−8∼−7.3)
Eastern Sub-Saharan Africa	465.19 (384.56∼547.84)	31.72 (26.31∼37.51)	721.1 (581.27∼878.7)	19.8 (15.94∼24.02)	−1.5 (−1.61∼−1.38)
High-income Asia Pacific	6.34 (5.51∼7.26)	0.35 (0.31∼0.4)	1.4 (1.15∼1.71)	0.09 (0.08∼0.11)	−4.81 (−5.02∼−4.6)
High-income North America	26.72 (22.39∼31.44)	0.88 (0.74∼1.04)	12.83 (10.53∼15.38)	0.39 (0.32∼0.47)	−2.4 (−2.7∼−2.09)
North Africa and Middle East	57.47 (50.61∼64.87)	1.75 (1.54∼1.98)	45.09 (36.58∼55.12)	0.66 (0.54∼0.81)	−3.42 (−3.5∼−3.34)
Oceania	4.21 (3.3∼5.2)	6.61 (5.24∼8.09)	7.98 (6.14∼10.39)	5.8 (4.44∼7.54)	−0.55 (−0.82∼−0.27)
South Asia	1212.98 (989.66∼1469.53)	11.18 (9.18∼13.49)	1063.07 (837.63∼1318.44)	5.33 (4.2∼6.6)	−2.8 (−3.1∼−2.49)
Southeast Asia	191.54 (162.92∼226.4)	4 (3.4∼4.73)	138.82 (114.42∼164.07)	1.91 (1.58∼2.26)	−2.78 (−3.03∼−2.52)
Southern Latin America	3.81 (3.14∼4.61)	0.77 (0.64∼0.93)	5 (4.14∼6.04)	0.74 (0.61∼0.89)	−0.08 (−0.42∼0.27)
Southern Sub-Saharan Africa	152.62 (129.31∼178.72)	29.38 (24.85∼34.39)	97.8 (72.62∼126.86)	11.14 (8.32∼14.39)	−1.97 (−3.08∼−0.85)
Tropical Latin America	56.07 (47.55∼65.86)	3.51 (2.99∼4.09)	27.89 (23.64∼32.96)	1.17 (0.99∼1.38)	−2.66 (−3.38∼−1.93)
Western Europe	13.89 (12.24∼15.95)	0.35 (0.31∼0.4)	5.72 (4.17∼7.7)	0.15 (0.11∼0.2)	−3.05 (−3.21∼−2.89)
Western Sub-Saharan Africa	629.75 (504.05∼795.72)	37.74 (30.26∼47.58)	1142.05 (881.71∼1489.9)	26.94 (20.75∼35.03)	−1.13 (−1.4∼−0.87)

DALY: disability-adjusted life years.

## Data Availability

In this study, the data of cervical cancer disease burden were obtained from the online Global Health Data Exchange (GHDx) query tool (https://ghdx.healthdata.org/gbdresults-tool).

## Abbreviations

GBD: The Global Burden of Disease
GCO: Global Cancer Observatory
GHDx: Global Health Data Exchange
SDG: Sustainable development goal
WHO: World Health Organization
ASIR: Age-standardized incidence rate
ASDR: Age-standardized death rate
ASR: Age-standardized incidence/death/DALY rate
DALY: Disability-adjusted life years
EAPC: Estimated annual percentage changes
SDI: Sociodemographic index
YLDs: The years lived with disability
YLLs: Years of life lost.

## Supplementary Materials

Supplementary Materials Supplementary Figure 1: the EAPC of ectopic pregnancy ASR from 1990 to 2019. (A). The EAPC of ASIR. (B). The EAPC of ASDR. (C). The EAPC of age-standardized DALY rate. Supplementary Figure 2: the relationships between age-standardized incidence (A), death (B), and DALY (C) rates of ectopic pregnancy per 100,000 population and SDI among different regions in 2019. Supplementary Figure 3: the global EAPC of ectopic pregnancy in 194 countries. (A). The EAPC for ASIR. (B). The EAPC of ASDR. (C). The EAPC of age-standardized DALY rate. Supplement Table S1: three countries with the largest and lowest number of incidence, death, or DALY. Supplement Table S2: three regions with the largest and lowest number of incidence, death, or DALY. Supplement Table S3: the incident cases and age-standardized incidence rate of ectopic pregnancy in 1990 and 2019, and its temporal trends from 1990 to 2019. Supplement Table S4: the death cases and age-standardized death rate of ectopic pregnancy in 1990 and 2019, and its temporal trends from 1990 to 2019. Supplement Table S5: the DALY and age-standardized DALY rate of ectopic pregnancy in 1990 and 2019, and its temporal trends from 1990 to 2019.
