# Supplementary material for: Global, Regional, and National Burden of Ectopic Pregnancy: A 30-Year Observational Database Study
Source: Int J Clin Pract. 2023 Dec 18;2023:3927337. doi: 10.1155/2023/3927337 (PMC10749725; doi:10.1155/2023/3927337)
Supplement: Supplementary Materials — Supplementary Figure 1: the EAPC of ectopic pregnancy ASR from 1990 to 2019. (A). The EAPC of ASIR. (B). The EAPC of ASDR. (C). The EAPC of age-standardized DALY rate. Supplementary Figure 2: the relationships between age-standardized incidence (A), death (B), and DALY (C) rates of ectopic pregnancy per 100,000 population and SDI among different regions in 2019. Supplementary Figure 3: the global EAPC of ectopic pregnancy in 194 countries. (A). The EAPC for ASIR. (B). The EAPC of ASDR. (C). The EAPC of age-standardized DALY rate. Supplement Table S1: three countries with the largest and lowest number of incidence, death, or DALY. Supplement Table S2: three regions with the largest and lowest number of incidence, death, or DALY. Supplement Table S3: the incident cases and age-standardized incidence rate of ectopic pregnancy in 1990 and 2019, and its temporal trends from 1990 to 2019. Supplement Table S4: the death cases and age-standardized death rate of ectopic pregnancy in 1990 and 2019, and its temporal trends from 1990 to 2019. Supplement Table S5: the DALY and age-standardized DALY rate of ectopic pregnancy in 1990 and 2019, and its temporal trends from 1990 to 2019. [file 3927337.f1.docx]

Supplement Table S1 Three countries with the largest and lowest number of incidence, death, or DALY.

| **Measure** | **Top three countries** | | | | **Bottom three countries** | | |
| --- | --- | --- | --- | --- | --- | --- | --- |
| **2019 ASR (per 100,000 population)** | |  |  | | | | |
| ASIR | Niger (444.61) | Papua New Guinea (407.76) | Chad (404.62) | Australia (30.1) | | South Africa (40.59) | Poland (47.27) |
| ASDR | Mauritania (1.93) | Chad (1.92) | Senegal (1.55) | Israel (0) | | Canada (0) | Greece (0) |
| Age standardized DALY Rate | Chad (108.11) | Mauritania (103.08) | Senegal (85.23) | Poland (0.12) | | Singapore (0.14) | Cyprus (0.15) |
| **1990-2019 increasing in the number of cases/years (-fold)** | | | | | | | |
| Incidence (cases) | Qatar (2.95) | Afghanistan (2.69) | Somalia (2.53) | Northern Mariana Islands (0.42) | | Albania (0.42) | Puerto Rico (0.43) |
| Death (cases) | Ecuador (11.54) | Belize (11.06) | Jamaica (9.96) | Estonia (0.01) | | Czechia (0.03) | Hungary (0.04) |
| DALY (years) | Belize (8.67) | Ecuador (8.19) | Haiti (7.7) | Poland (0.07) | | Hungary (0.08) | Estonia (0.08) |
| **EAPC** | | | | | | | |
| Incidence | Qatar (2.95) | Afghanistan (2.69) | Somalia (2.53) | Northern Mariana Islands (0.42) | | Albania (0.42) | Puerto Rico (0.43) |
| Death | Ecuador (11.54) | Belize (11.06) | Jamaica (9.96) | Estonia (0.01) | | Czechia (0.03) | Hungary (0.04) |
| DALY | Belize (8.67) | Ecuador (8.19) | Haiti (7.7) | Poland (0.07) | | Hungary (0.08) | Estonia (0.08) |

Supplement Table S2 Three regions with the largest and lowest number of incidence, death, or DALY.

| **Measure** | **Top three regions** | | | **Bottom three regions** | | |
| --- | --- | --- | --- | --- | --- | --- |
| **2019 ASR (per 100,000 population)** | |  |  | | | |
| ASIR | Oceania (187.22) | Eastern Europe (173.85) | Southern Latin America (169.79) | High-income Asia Pacific (29.81) | Australasia (33.22) | High-income North America (39.7) |
| ASDR | Central Sub-Saharan Africa (0.55) | Western Sub-Saharan Africa (0.48) | Eastern Sub-Saharan Africa (0.37) | Eastern Europe (0) | Central Asia (0) | Western Europe (0) |
| Age standardized DALY Rate | Central Sub-Saharan Africa (30.58) | Western Sub-Saharan Africa (26.94) | Eastern Sub-Saharan Africa (19.8) | High-income Asia Pacific (0.09) | Central Europe (0.12) | Australasia (0.14) |
| **1990-2019 increasing in the number of cases/years (-fold)** | | | | | | |
| Incidence (cases) | Oceania (1.96) | Western Sub-Saharan Africa (1.8) | Central Sub-Saharan Africa (1.66) | East Asia (0.57) | High-income North America (0.68) | Central Europe (0.7) |
| Death (cases) | Caribbean (5.9) | Andean Latin America (3.6) | Central Sub-Saharan Africa (2.23) | Eastern Europe (0.08) | Central Europe (0.1) | East Asia (0.16) |
| DALY (years) | Caribbean (5.45) | Andean Latin America (3.22) | Central Sub-Saharan Africa (2.21) | Eastern Europe (0.12) | Central Europe (0.16) | East Asia (0.19) |
| **EAPC** | | | | | | |
| Incidence | Oceania (1.96) | Western Sub-Saharan Africa (1.8) | Central Sub-Saharan Africa (1.66) | East Asia (0.57) | High-income North America (0.68) | Central Europe (0.7) |
| Death | Caribbean (5.9) | Andean Latin America (3.6) | Central Sub-Saharan Africa (2.23) | Eastern Europe (0.08) | Central Europe (0.1) | East Asia (0.16) |
| DALY | Caribbean (5.45) | Andean Latin America (3.22) | Central Sub-Saharan Africa (2.21) | Eastern Europe (0.12) | Central Europe (0.16) | East Asia (0.19) |

Supplement Table S3 The incident cases and age-standardized incidence rate of ectopic pregnancy in 1990 and 2019, and its temporal trends from 1990 to 2019.

| **Nation** | **Incident cases No. 10^2^ (95% UI)** | | **Change in absolute  number (%)** | **ASIR per 100,000 No. 10^2^ (95% UI)** | | **1990-2019 EAPC No. (95% CI)** |
| --- | --- | --- | --- | --- | --- | --- |
|  | **1990** | **2019** |  | **1990** | **2019** |  |
| Afghanistan | 168.55 (126.7~225.44) | 452.59 (339.58~602.91) | 168.52 | 370.38 (277.07~500.02) | 269.84 (202.53~362.5) | -1.15 (-1.28~-1.02) |
| Albania | 50.33 (36.12~68.91) | 21.21 (15.42~28.48) | -57.86 | 272.96 (197.06~368.57) | 166.47 (121.31~223.15) | -1.78 (-2.1~-1.45) |
| Algeria | 267.78 (195.22~357.88) | 411.69 (295.14~568.1) | 53.74 | 251.83 (183.73~339.96) | 169.38 (121.62~233.55) | -0.64 (-1.21~-0.07) |
| American Samoa | 0.96 (0.71~1.3) | 0.59 (0.44~0.79) | -38.54 | 384.22 (284.36~518.72) | 234.74 (171.53~315.75) | -1.69 (-1.73~-1.64) |
| Andorra | 0.49 (0.35~0.68) | 0.57 (0.41~0.78) | 16.33 | 147.44 (107.55~204.61) | 144.57 (103.13~195.81) | -0.01 (-0.05~0.03) |
| Angola | 201.63 (151.92~269.32) | 429.67 (324.11~585.28) | 113.1 | 436.7 (331.33~585.1) | 300.83 (227.28~406.18) | -1.22 (-1.36~-1.08) |
| Antigua and Barbuda | 0.44 (0.32~0.6) | 0.42 (0.3~0.57) | -4.55 | 125.18 (92.74~169.55) | 87.37 (63.88~118.37) | -1.44 (-1.53~-1.36) |
| Argentina | 745.51 (557.91~989.2) | 835.61 (615.98~1133.26) | 12.09 | 465.49 (347.47~618.37) | 352.47 (259.65~474.04) | -0.78 (-0.87~-0.7) |
| Armenia | 44.41 (32.13~59.71) | 23.64 (17.37~31.78) | -46.77 | 232.03 (169.37~309.11) | 155.76 (114.72~207.34) | -0.86 (-1.37~-0.36) |
| Australia | 46.55 (33.45~65.98) | 36.31 (28.36~46.9) | -22 | 51.09 (36.71~72.28) | 30.1 (23.56~38.99) | -0.58 (-1.22~0.05) |
| Austria | 153.95 (117.7~198.64) | 159.09 (118.89~211.57) | 3.34 | 375.02 (289.36~478.99) | 393.08 (295.25~522.52) | -0.02 (-0.25~0.2) |
| Azerbaijan | 114.63 (83.74~155.67) | 82.47 (59.93~110.92) | -28.06 | 260.8 (192.69~350.81) | 149.56 (110.14~202.48) | -1.32 (-1.59~-1.05) |
| Bahamas | 2.07 (1.52~2.82) | 1.8 (1.33~2.42) | -13.04 | 132.67 (98.44~178.51) | 86.53 (63.57~115.33) | -1.79 (-1.95~-1.64) |
| Bahrain | 4.27 (3.12~5.96) | 5.67 (4.09~7.84) | 32.79 | 170.11 (125.64~232.69) | 81.85 (59.88~112.8) | -2.62 (-2.84~-2.4) |
| Bangladesh | 1497.02 (1110.17~2029.11) | 1054.65 (775.6~1429.09) | -29.55 | 275.36 (205.8~368.18) | 112.19 (83.12~151.81) | -2.94 (-3.03~-2.86) |
| Barbados | 1.6 (1.18~2.15) | 1.19 (0.88~1.59) | -25.63 | 109.5 (81.91~147.09) | 86.5 (63.97~116.76) | -0.72 (-0.93~-0.5) |
| Belarus | 154.54 (111.16~208.24) | 142.65 (101.11~195.57) | -7.69 | 299.78 (217.43~402.25) | 323.57 (229.76~442.83) | 1.27 (0.75~1.79) |
| Belgium | 115.52 (81.97~157.53) | 125.81 (90.89~170.06) | 8.91 | 228.66 (163.06~310.58) | 251.62 (180.92~337.09) | 0.41 (0.32~0.51) |
| Belize | 2.35 (1.76~3.1) | 2.91 (2.16~3.97) | 23.83 | 272.3 (205.4~358.21) | 125.49 (92.96~169.92) | -2.51 (-2.61~-2.41) |
| Benin | 92.59 (70.87~125.47) | 189.91 (141.67~256.89) | 105.11 | 416.8 (319.53~565.05) | 311.07 (233.39~415.21) | -0.87 (-0.92~-0.83) |
| Bermuda | 0.35 (0.25~0.5) | 0.26 (0.19~0.37) | -25.71 | 94.76 (69.3~131.9) | 89.7 (64~123.95) | 0.01 (-0.27~0.29) |
| Bhutan | 9.21 (6.88~12.45) | 5.55 (4.04~7.58) | -39.74 | 314.95 (235.89~421.02) | 127.7 (93.14~174.86) | -3.26 (-3.33~-3.2) |
| Bolivia | 161.4 (121.23~213.91) | 217.82 (161.86~291.96) | 34.96 | 537.91 (404.37~714.52) | 351.68 (261.72~471.56) | -1.35 (-1.53~-1.17) |
| Bosnia and Herzegovina | 37.3 (26.89~51.34) | 15.88 (11.3~21.51) | -57.43 | 151.7 (109.87~208.43) | 112.43 (79.32~151.82) | -1.15 (-1.58~-0.72) |
| Botswana | 33.93 (25.71~43.6) | 41.28 (30.89~53.45) | 21.66 | 524.44 (395.53~672.49) | 297.37 (224.68~385.61) | -1.81 (-1.87~-1.74) |
| Brazil | 960.56 (738.79~1258.8) | 1004.07 (752.83~1364.54) | 4.53 | 117.89 (91.11~154.41) | 85.44 (64.41~115.48) | -0.93 (-1.04~-0.82) |
| Brunei Darussalam | 2.31 (1.71~3.06) | 2.17 (1.58~2.97) | -6.06 | 153.45 (114.2~205.16) | 83.5 (61.32~114.99) | -2.21 (-2.34~-2.08) |
| Bulgaria | 52.08 (37.27~70.08) | 35.97 (26.59~48.27) | -30.93 | 139.97 (99.56~189.07) | 135.63 (99.39~183.58) | 0.8 (0.45~1.15) |
| Burkina Faso | 182.85 (139.49~246.15) | 356.66 (267.08~486.19) | 95.06 | 438.13 (334.88~587.28) | 330.32 (250.08~446.31) | -1.07 (-1.11~-1.03) |
| Burundi | 105.73 (78.69~144.57) | 187.42 (139.43~250.01) | 77.26 | 432.43 (319.94~593.54) | 355.59 (262.37~471.35) | -0.62 (-0.69~-0.54) |
| Cambodia | 149.91 (112.54~200.09) | 114.48 (83.28~156.75) | -23.63 | 302.91 (226.39~403.36) | 122.51 (89.98~168.08) | -3.32 (-3.52~-3.12) |
| Cameroon | 172.27 (130.32~236.16) | 329.39 (245.43~454.85) | 91.21 | 357.02 (271.86~481.43) | 218.18 (163.94~297.86) | -1.41 (-1.66~-1.16) |
| Canada | 173.03 (123.76~237.96) | 184.6 (132.13~263.56) | 6.69 | 110.62 (79.5~151.78) | 111.18 (80.2~158.5) | 0.32 (0.17~0.47) |
| Cape Verde | 4.27 (3.18~5.77) | 3.77 (2.81~5.14) | -11.71 | 268.02 (202.13~359.11) | 121.89 (91.33~165.34) | -2.83 (-2.97~-2.68) |
| Central African Republic | 48.45 (36.6~65.78) | 74.17 (56.21~99.66) | 53.09 | 371.4 (280.16~499.91) | 275.37 (206.48~368.62) | -0.92 (-1.01~-0.82) |
| Chad | 122.51 (92.52~166.38) | 291.6 (222.86~392.1) | 138.02 | 450.48 (343.35~613.27) | 404.62 (308.89~542.11) | -0.31 (-0.34~-0.29) |
| Chile | 317.28 (234.14~424.23) | 281.22 (205.86~380.11) | -11.37 | 419.39 (312.14~561.49) | 299.07 (220.13~402.12) | -1.04 (-1.24~-0.83) |
| China | 24721.74 (18385.75~32560.05) | 14144.17 (10730.76~19052.73) | -42.79 | 356.04 (261.83~470.8) | 192 (145.74~253.05) | -0.82 (-1.31~-0.32) |
| Colombia | 397.47 (299.12~527.22) | 346.35 (260.2~456.5) | -12.86 | 222.98 (169.33~293.88) | 136.51 (102.93~179.98) | -1.81 (-1.86~-1.76) |
| Comoros | 8.02 (6.11~10.82) | 6.65 (4.94~9.05) | -17.08 | 399.25 (299.99~542.31) | 180.84 (133.42~245.21) | -2.76 (-2.86~-2.67) |
| Cook Islands | 0.27 (0.2~0.36) | 0.16 (0.12~0.22) | -40.74 | 301.56 (225.91~410.76) | 198.32 (143.09~267.83) | -1.46 (-1.47~-1.44) |
| Costa Rica | 33.69 (25.33~45.06) | 29.53 (21.66~39.02) | -12.35 | 206.29 (156.5~276.2) | 110.9 (81.96~146.38) | -2.05 (-2.33~-1.77) |
| Croatia | 30.37 (21.56~41.04) | 22.43 (16.51~30.19) | -26.14 | 130.88 (92.82~178.38) | 123.21 (90.72~166.75) | -0.1 (-0.36~0.15) |
| Cuba | 57.9 (42.27~80.02) | 40.66 (29.41~55.67) | -29.78 | 89.22 (65.96~122.64) | 84.69 (61.43~115.31) | 0.3 (0.09~0.51) |
| Cyprus | 9.1 (6.54~12.18) | 7.38 (5.24~10.29) | -18.9 | 225.74 (161.77~300.64) | 93.26 (66.46~129.73) | -2.97 (-3.34~-2.6) |
| Czech Republic | 92.17 (66.81~124.6) | 89.29 (63.73~121.88) | -3.12 | 205.42 (147.15~278.13) | 200.87 (144.34~274) | 0.89 (0.35~1.44) |
| Democratic Republic of the Congo | 741.88 (564.16~1014.24) | 1164.71 (866.23~1585.06) | 56.99 | 441.34 (335.14~591.71) | 286.07 (212.76~391.21) | -1.31 (-1.53~-1.08) |
| Denmark | 58.36 (41.41~78.61) | 62.92 (45.12~85.88) | 7.81 | 231.27 (163.95~311.25) | 257.02 (183.73~351.69) | 0.29 (0.25~0.34) |
| Djibouti | 8.68 (6.53~11.63) | 14.8 (11~20.04) | 70.51 | 423.33 (321.59~563.75) | 229.24 (171.58~308.87) | -2.16 (-2.35~-1.98) |
| Dominica | 0.61 (0.45~0.82) | 0.35 (0.26~0.48) | -42.62 | 173.44 (128.76~235.86) | 106.08 (78.23~145.04) | -1.89 (-1.96~-1.82) |
| Dominican Republic | 85.15 (63.08~113.68) | 82.09 (60.12~111.7) | -3.59 | 212.4 (159.41~282.66) | 138.77 (102.57~188.37) | -1.54 (-1.72~-1.36) |
| Ecuador | 169.35 (129.14~223.63) | 246.19 (189.61~313.02) | 45.37 | 333.18 (254.04~436.06) | 263.74 (203.64~334.92) | -0.31 (-0.52~-0.1) |
| Egypt | 658.27 (485.37~882.74) | 690.26 (508.67~935.79) | 4.86 | 246.68 (183.14~328.54) | 132.02 (97.22~178.39) | -2.12 (-2.75~-1.49) |
| El Salvador | 66.27 (50.03~87.61) | 48.31 (36.43~65.28) | -27.1 | 254.28 (193.1~334.16) | 136.77 (103.14~183.3) | -2.27 (-2.56~-1.99) |
| Equatorial Guinea | 9.07 (6.9~11.97) | 13.97 (10.55~19.09) | 54.02 | 458.95 (349.96~607.65) | 198.71 (150.9~267.97) | -3.01 (-3.22~-2.81) |
| Eritrea | 53.75 (40.98~72.25) | 79.56 (59.51~107.26) | 48.02 | 406.53 (307.34~544.08) | 242.9 (183.25~326.37) | -1.83 (-1.97~-1.69) |
| Estonia | 24.32 (17.63~33.61) | 20.5 (14.44~28.3) | -15.71 | 332.09 (238.8~457.27) | 341.32 (241.71~467.77) | 1.21 (0.81~1.62) |
| Eswatini | 10.56 (8.01~14.26) | 9.42 (7.13~12.81) | -10.8 | 264.2 (200.35~357.34) | 142.52 (108.26~193.51) | -2.09 (-2.12~-2.07) |
| Ethiopia | 1049.18 (800.33~1394.48) | 1520.54 (1145.46~2035.97) | 44.93 | 461.84 (351.52~612.17) | 292.43 (219.94~392.28) | -1.67 (-1.77~-1.58) |
| Fiji | 9.82 (7.26~13.21) | 9.98 (7.14~13.64) | 1.63 | 240.03 (178.08~319.95) | 215.51 (154.16~294.35) | -0.28 (-0.34~-0.22) |
| Finland | 52.77 (38.66~72.55) | 43.32 (31.12~59.64) | -17.91 | 211.13 (155.19~289.69) | 185.06 (133.65~254.93) | -0.55 (-0.74~-0.37) |
| France | 565.02 (405.74~781.99) | 594.57 (427.67~823.01) | 5.23 | 192.31 (137.57~267.59) | 214.34 (155.02~295.28) | 0.57 (0.42~0.73) |
| Gabon | 15.24 (11.46~20.5) | 16.09 (12.11~21.82) | 5.58 | 342.65 (260.17~453.41) | 162.71 (121.88~219.6) | -2.52 (-2.56~-2.49) |
| Gambia | 19.06 (14.56~26.09) | 28.01 (21.04~37.63) | 46.96 | 423.5 (326.11~569.98) | 247.2 (186.49~334.03) | -1.87 (-2.01~-1.73) |
| Georgia | 46.25 (33.98~61.91) | 29.57 (22.98~38.51) | -36.06 | 161.87 (119.72~214.98) | 181.17 (140.61~234.63) | 1.24 (0.87~1.6) |
| Germany | 293.36 (209.86~408.75) | 283.69 (251.62~324.09) | -3.3 | 71.08 (51.14~98.77) | 79.78 (70.81~91.09) | 0.88 (0.71~1.05) |
| Ghana | 235.68 (180.1~315.53) | 333.39 (249.53~457.55) | 41.46 | 340.52 (259.7~451.78) | 190.11 (142.67~260.54) | -1.87 (-2~-1.74) |
| Greece | 72.69 (53.12~97.61) | 74.85 (52.73~103.54) | 2.97 | 146.03 (106.87~196.32) | 168.05 (117.86~232.97) | 0.87 (0.71~1.02) |
| Greenland | 0.49 (0.35~0.67) | 0.34 (0.25~0.46) | -30.61 | 145.13 (108.32~196.59) | 123.93 (91.62~167.53) | -0.72 (-0.83~-0.62) |
| Grenada | 0.82 (0.61~1.12) | 0.62 (0.46~0.83) | -24.39 | 204.49 (153.55~281.44) | 120.95 (90.66~162.26) | -1.58 (-1.84~-1.32) |
| Guam | 1.86 (1.4~2.53) | 1.82 (1.33~2.44) | -2.15 | 246.11 (185.72~334.85) | 244.15 (178.11~327.43) | -0.18 (-0.37~0.01) |
| Guatemala | 160.36 (122.61~212.73) | 182.96 (138.85~243.41) | 14.09 | 464.48 (353.26~615.38) | 180.94 (136.51~239.17) | -3.57 (-3.8~-3.34) |
| Guinea | 120.35 (91.34~162.75) | 179.19 (137.31~240.75) | 48.89 | 418.85 (316.67~563.9) | 281.39 (214.17~380.24) | -1.45 (-1.48~-1.41) |
| Guinea-Bissau | 18.83 (14.27~25.18) | 25.34 (19.04~34.19) | 34.57 | 404.11 (306.83~542.26) | 258.25 (194.79~345.67) | -1.58 (-1.68~-1.47) |
| Guyana | 7.68 (5.69~10.26) | 5.22 (3.88~6.98) | -32.03 | 174.42 (130.53~231.87) | 121.92 (91.11~163.17) | -1.09 (-1.15~-1.02) |
| Haiti | 114.96 (85.82~152.04) | 156.51 (113.72~214.95) | 36.14 | 385.83 (287.61~510.36) | 221.78 (162.21~301.98) | -1.94 (-1.95~-1.92) |
| Honduras | 80.47 (61.72~106.28) | 97.43 (73.35~129.82) | 21.08 | 395.65 (300.03~517.97) | 176.43 (133.29~236.91) | -3.07 (-3.27~-2.86) |
| Hungary | 69.76 (50.43~94.86) | 51.98 (37.65~69.25) | -25.49 | 156.05 (112.72~215.47) | 126.94 (91.82~170.89) | -0.3 (-0.62~0.03) |
| Iceland | 3.39 (2.45~4.59) | 3.15 (2.28~4.36) | -7.08 | 247.68 (180.29~334.56) | 195.34 (141.94~271.23) | -0.53 (-0.75~-0.31) |
| India | 11563.32 (8785.91~15373.32) | 9308.22 (6816.62~12607.36) | -19.5 | 273.63 (209.16~361.83) | 122.25 (89.78~165.19) | -2.85 (-2.9~-2.79) |
| Indonesia | 1333.99 (993.13~1802.83) | 1194.84 (900.71~1620.6) | -10.43 | 135.93 (102.13~183.69) | 85.6 (64.6~116.1) | -1.37 (-1.5~-1.24) |
| Iran | 317.51 (241.83~426.23) | 267.99 (196.56~383.82) | -15.6 | 123.53 (93.78~170.1) | 52.5 (38.9~73.41) | -2.38 (-2.82~-1.94) |
| Iraq | 240.1 (183.33~314.74) | 311.59 (232.65~422.11) | 29.78 | 326.2 (245.44~430.9) | 138.48 (102.96~187.46) | -3.11 (-3.19~-3.03) |
| Ireland | 43.26 (31.45~58.74) | 54.53 (39.22~75.18) | 26.05 | 247.66 (179.88~336.92) | 221.95 (163.4~303.01) | -0.01 (-0.16~0.14) |
| Israel | 78.51 (57.83~107.82) | 156.52 (112.77~214.31) | 99.36 | 320.39 (234.85~439.27) | 366.15 (263.76~501.65) | 0.39 (0.29~0.5) |
| Italy | 263.42 (169.5~393.32) | 351.14 (259.19~463.2) | 33.3 | 92.35 (59.5~137.76) | 144.47 (106.46~190.36) | 1.61 (1.21~2) |
| Jamaica | 19.75 (14.75~26.53) | 15.07 (11.14~20.54) | -23.7 | 155.93 (116.34~212.52) | 95.2 (70.67~129.92) | -1.76 (-1.84~-1.68) |
| Japan | 412.48 (294.26~575.89) | 325.1 (235.61~455.15) | -21.18 | 74.04 (52.68~104.03) | 69.28 (50.29~96.12) | -0.04 (-0.21~0.14) |
| Jordan | 56.54 (41.68~76.22) | 119.61 (89.87~157.43) | 111.55 | 368.62 (267.35~499.25) | 209.41 (156.8~276.8) | -1.69 (-1.87~-1.5) |
| Kazakhstan | 221.5 (165.57~296.7) | 231.62 (169.62~315.23) | 4.57 | 249.83 (187.3~333.55) | 237.25 (173.67~320.64) | 0.78 (0.34~1.22) |
| Kenya | 416.74 (320.22~564.39) | 507.6 (384.57~686.1) | 21.8 | 400.14 (307.34~530.61) | 183.49 (138.53~247.39) | -2.6 (-2.64~-2.55) |
| Kiribati | 1.51 (1.11~2.05) | 1.84 (1.34~2.52) | 21.85 | 395.59 (291.69~536.97) | 288.23 (210.45~392.65) | -1.03 (-1.11~-0.96) |
| Kuwait | 9.59 (7.19~12.67) | 15.18 (11.04~20.92) | 58.29 | 106.88 (80.83~140.77) | 47.86 (35.12~64.91) | -3.89 (-4.73~-3.04) |
| Kyrgyzstan | 76.14 (56~101.94) | 91.1 (67.1~122.95) | 19.65 | 333.67 (246.79~443.55) | 255.61 (188.64~342.89) | 0.06 (-0.37~0.5) |
| Laos | 53.25 (40.05~69.53) | 52.64 (39.03~70.42) | -1.15 | 281.53 (211.71~368.33) | 130.68 (96.91~175.12) | -3.06 (-3.24~-2.89) |
| Latvia | 45.74 (33.39~61.78) | 32.21 (23.1~44.2) | -29.58 | 363.81 (264.04~494.92) | 383.05 (276~519.24) | 1.06 (0.51~1.62) |
| Lebanon | 34.81 (26.09~45.98) | 29.73 (22.08~39.98) | -14.59 | 225.11 (169.41~299.43) | 104.15 (77.65~139.03) | -2.55 (-2.62~-2.49) |
| Lesotho | 21.32 (16.21~28.72) | 15.67 (11.62~20.79) | -26.5 | 248.63 (190.38~334.51) | 132.67 (99.59~177.64) | -2.02 (-2.12~-1.91) |
| Liberia | 35.1 (26.63~47.51) | 51.26 (38.02~69.84) | 46.04 | 402.36 (304.99~545.19) | 204.33 (153.33~276.51) | -2.33 (-2.5~-2.16) |
| Libya | 40.95 (30.84~55.01) | 38.06 (27.3~51.5) | -7.06 | 259.92 (195.62~351.88) | 91.62 (65.95~123.97) | -3.18 (-3.38~-2.98) |
| Lithuania | 57.81 (42~77.61) | 36.4 (26.13~50.43) | -37.04 | 311.97 (227.23~416.35) | 302.26 (217.13~420.77) | 0.56 (0.03~1.1) |
| Luxembourg | 4.43 (3.09~5.97) | 6.9 (4.87~9.42) | 55.76 | 211.81 (148.7~284.01) | 214.08 (150.48~289.47) | -0.13 (-0.23~-0.02) |
| Madagascar | 204.09 (151.33~274.07) | 318.03 (239.98~436.57) | 55.83 | 378.23 (281.38~510.19) | 231.66 (175.44~316.13) | -1.69 (-1.79~-1.6) |
| Malawi | 187.6 (141.98~251.69) | 222.86 (164.41~300.39) | 18.8 | 430.36 (327.57~569.63) | 238.2 (180.31~316.19) | -2.12 (-2.33~-1.92) |
| Malaysia | 171.67 (126.59~234.1) | 201.12 (145.77~277.87) | 17.16 | 186.95 (137.9~252.85) | 115.71 (83.81~159.22) | -2.01 (-2.18~-1.84) |
| Maldives | 2.24 (1.67~3.01) | 2.67 (1.93~3.68) | 19.2 | 251.97 (187.81~336.75) | 104.72 (76.79~142.42) | -2.69 (-3.07~-2.3) |
| Mali | 178.25 (135.02~238.24) | 364.37 (275.77~489.66) | 104.42 | 463.49 (352.53~611.33) | 367.74 (280.7~487.36) | -0.85 (-0.89~-0.81) |
| Malta | 1.9 (1.37~2.59) | 1.55 (1.11~2.2) | -18.42 | 102.3 (73.75~141.21) | 77.95 (55.93~108.67) | -1.01 (-1.52~-0.49) |
| Marshall Islands | 0.75 (0.56~1.01) | 0.69 (0.51~0.93) | -8 | 365.57 (270.12~486.59) | 229.38 (170.14~310.22) | -1.36 (-1.47~-1.25) |
| Mauritania | 36.5 (27.53~49.3) | 44.32 (33.19~59.82) | 21.42 | 395.66 (298.34~534.67) | 226.39 (168.16~306.96) | -1.73 (-1.88~-1.59) |
| Mauritius | 6.6 (4.87~8.98) | 4.24 (3.1~5.75) | -35.76 | 101.03 (74.71~136.27) | 67.2 (49.21~91.37) | -1.55 (-1.63~-1.47) |
| Mexico | 889.09 (630.33~1244.66) | 1069.46 (834.51~1390.83) | 20.29 | 202.86 (142.75~287.02) | 158.21 (123.36~205.48) | -1.75 (-2.04~-1.46) |
| Micronesia | 2.2 (1.63~2.93) | 1.17 (0.85~1.57) | -46.82 | 508 (377.72~679.62) | 236.53 (169.84~319.75) | -2.6 (-2.69~-2.5) |
| Moldova | 89.56 (64.9~120.01) | 42.24 (29.88~56.31) | -52.84 | 390.79 (285.32~519.43) | 232.81 (167.52~306.78) | -1.25 (-1.57~-0.94) |
| Monaco | 0.25 (0.18~0.34) | 0.24 (0.17~0.34) | -4 | 183.72 (134.74~251.09) | 177.78 (126.2~248) | -0.11 (-0.15~-0.07) |
| Mongolia | 45.73 (33.92~61.48) | 57.39 (42.07~78.21) | 25.5 | 428.03 (321.89~564.34) | 302.95 (224.76~408.75) | -0.27 (-0.91~0.38) |
| Montenegro | 5.49 (3.91~7.45) | 4.06 (2.88~5.52) | -26.05 | 172.28 (123.05~233.48) | 143.75 (102.05~194.85) | -0.66 (-0.72~-0.59) |
| Morocco | 279.58 (208.32~379.35) | 237.58 (175.36~320.8) | -15.02 | 230.5 (170.77~312.49) | 121.45 (89.87~163.72) | -2.19 (-2.25~-2.14) |
| Mozambique | 236.45 (182.9~314.99) | 418.6 (316.57~556.35) | 77.04 | 384.75 (296.72~507.82) | 293.09 (223.67~388.65) | -0.85 (-0.98~-0.71) |
| Myanmar | 434.13 (331.38~572.74) | 359.03 (267.9~482.61) | -17.3 | 213.91 (162.03~281.07) | 119.58 (89.14~160.59) | -1.93 (-1.97~-1.88) |
| Namibia | 16.59 (12.42~22.16) | 20.71 (15.68~28.1) | 24.83 | 254.53 (192.3~339.6) | 156.72 (117.56~211.87) | -1.51 (-1.6~-1.42) |
| Nauru | 0.25 (0.18~0.34) | 0.16 (0.12~0.22) | -36 | 500.75 (364.69~684.33) | 289.32 (213.53~390.31) | -1.98 (-2.02~-1.94) |
| Nepal | 338.62 (252.03~447.17) | 228.54 (161.18~311.42) | -32.51 | 352.32 (266.18~466.57) | 119.29 (84.86~161.97) | -4.12 (-4.3~-3.94) |
| Netherlands | 149.85 (105.32~208.48) | 143.63 (99.42~200.06) | -4.15 | 183.62 (128.97~255.2) | 198.49 (137.46~275.93) | 0.43 (0.3~0.56) |
| New Zealand | 43.56 (27.09~68.84) | 55.08 (41.86~72.23) | 26.45 | 235.43 (147.46~369.72) | 281.82 (214.4~369.58) | -0.12 (-0.34~0.09) |
| Nicaragua | 59.74 (44.74~80.38) | 54.19 (41.08~71.69) | -9.29 | 326.69 (246.26~438.56) | 148.32 (112.64~195.45) | -2.57 (-2.93~-2.21) |
| Niger | 170.48 (129.89~227.56) | 424.81 (319.33~570.43) | 149.18 | 485 (373.73~645.85) | 444.61 (334.52~595.95) | -0.3 (-0.37~-0.24) |
| Nigeria | 1813.81 (1396.54~2398.83) | 3288.26 (2531.34~4366.68) | 81.29 | 463.29 (357.87~612.11) | 318.31 (242.43~421.56) | -1.25 (-1.38~-1.13) |
| Niue | 0.03 (0.03~0.05) | 0.02 (0.01~0.02) | -33.33 | 348.18 (260.99~476.69) | 218.41 (158.94~290.44) | -1.65 (-1.68~-1.61) |
| North Korea | 607.59 (440.78~828.78) | 275.04 (195.44~373.75) | -54.73 | 550.77 (399.49~752.14) | 208.26 (148.69~282.71) | -3.26 (-3.34~-3.18) |
| North Macedonia | 19.04 (13.76~26.17) | 12.49 (8.92~17.13) | -34.4 | 182.49 (131.91~250.93) | 119.68 (84.82~163.42) | -1.48 (-1.71~-1.25) |
| Northern Mariana Islands | 0.65 (0.48~0.89) | 0.28 (0.21~0.37) | -56.92 | 210.4 (156.19~283.24) | 176.63 (128.74~238.38) | -0.39 (-0.46~-0.33) |
| Norway | 34.54 (21.54~52.69) | 37.07 (22.51~58.58) | 7.32 | 164.65 (102.41~250.36) | 151.74 (92.47~236.77) | -0.08 (-0.24~0.08) |
| Oman | 25.15 (18.72~34.58) | 25.61 (18.44~35.34) | 1.83 | 374.78 (278.83~509.26) | 116.18 (84.11~159.42) | -4.27 (-4.46~-4.08) |
| Pakistan | 2216.98 (1694.47~2963.28) | 2922.45 (2198.64~3911.07) | 31.82 | 463.99 (354.51~614.65) | 253.31 (191.22~335.39) | -2.11 (-2.26~-1.96) |
| Palau | 0.21 (0.15~0.27) | 0.12 (0.09~0.16) | -42.86 | 241.86 (180.56~322.11) | 169.84 (123.39~231.3) | -1.2 (-1.25~-1.16) |
| Palestine | 29 (21.59~38.96) | 34.53 (25.74~46.29) | 19.07 | 337.72 (249.64~460.96) | 131.98 (99.05~177.51) | -3.42 (-3.67~-3.17) |
| Panama | 23.24 (17.39~31.55) | 32.61 (24.39~43.38) | 40.32 | 183.62 (136.85~245.65) | 157.61 (117.83~209.54) | -0.45 (-0.59~-0.31) |
| Papua New Guinea | 88.85 (66.01~118.72) | 205 (153.23~273.68) | 130.73 | 476.02 (352~630.99) | 407.76 (303.16~544.52) | -0.58 (-0.6~-0.55) |
| Paraguay | 30.48 (23.08~40.96) | 31.07 (22.68~42.85) | 1.94 | 160.05 (121.59~217.35) | 81.37 (59.54~111.57) | -2.29 (-2.33~-2.24) |
| Peru | 428.47 (323.02~569.7) | 431.14 (317.39~583.91) | 0.62 | 399.6 (302.8~531.27) | 237.41 (175.54~320.6) | -1.74 (-1.83~-1.64) |
| Philippines | 926.67 (699.8~1234.42) | 1123.33 (847.11~1505.82) | 21.22 | 302.25 (228.31~403.5) | 194.36 (146.89~260.19) | -1.51 (-1.58~-1.45) |
| Poland | 113.93 (81.91~159.14) | 84.08 (71.01~100.8) | -26.2 | 64.05 (45.43~89.95) | 47.27 (39.96~56.41) | -0.8 (-1.24~-0.36) |
| Portugal | 53.42 (38.64~74.02) | 43.3 (32.4~57.6) | -18.94 | 107.29 (77.46~149.04) | 95.04 (71.33~127.18) | -0.32 (-0.4~-0.24) |
| Puerto Rico | 22.7 (16.6~30.67) | 9.66 (7.16~12.96) | -57.44 | 118.47 (86.56~160.37) | 62.54 (46.06~84.6) | -2.35 (-2.57~-2.13) |
| Qatar | 4.15 (3~5.61) | 12.26 (8.71~16.65) | 195.42 | 236.38 (172.96~318.11) | 108.33 (78.62~147.82) | -2.78 (-2.84~-2.72) |
| Republic of Congo | 39.21 (29.38~52.64) | 57.49 (42.83~77.74) | 46.62 | 350.01 (266.02~463.16) | 207.3 (154.54~280.95) | -1.44 (-1.6~-1.28) |
| Romania | 205.06 (147.52~270.38) | 139.22 (103.59~185.92) | -32.11 | 184.66 (132.95~245.97) | 186.61 (136.44~248.52) | 0.54 (0.2~0.89) |
| Russia | 2434.19 (1843.9~3197.31) | 2618.97 (1951.28~3505.54) | 7.59 | 329.08 (250.91~429.2) | 372.05 (281.06~485.62) | 1.74 (1.13~2.36) |
| Rwanda | 126.31 (94.46~169.96) | 139.15 (103.22~188.72) | 10.17 | 412.57 (311.36~551.44) | 212.63 (157.2~285.51) | -2.59 (-2.74~-2.44) |
| Saint Helena | 221.19 (165.29~294.27) | 342.71 (260.51~470.25) | 54.94 | 402.55 (303.36~537.99) | 262.24 (200.22~356.48) | -1.44 (-1.46~-1.41) |
| Saint Kitts and Nevis | 0.33 (0.24~0.44) | 0.28 (0.21~0.38) | -15.15 | 150.73 (112.95~200.94) | 90.96 (67.83~123.14) | -1.77 (-1.8~-1.74) |
| Saint Lucia | 1.24 (0.92~1.69) | 0.77 (0.58~1.05) | -37.9 | 173.4 (129.53~233.99) | 84.32 (62.61~113.79) | -2.51 (-2.67~-2.36) |
| Saint Vincent | 0.92 (0.68~1.25) | 0.62 (0.45~0.84) | -32.61 | 165.44 (122.83~223.27) | 111.92 (82.12~152.04) | -1.33 (-1.47~-1.2) |
| Samoa | 1.63 (1.19~2.21) | 1.14 (0.82~1.55) | -30.06 | 231.06 (168.08~313.35) | 120.26 (86.34~164.29) | -2.25 (-2.35~-2.15) |
| San Marino | 0.2 (0.15~0.28) | 0.27 (0.2~0.37) | 35 | 172.54 (124.5~238.61) | 177.6 (127.79~241.08) | 0.1 (0.08~0.12) |
| Sao Tome and Principe | 1.82 (1.35~2.45) | 1.85 (1.4~2.46) | 1.65 | 364.95 (272.67~487.64) | 174.22 (131.08~232.06) | -2.61 (-2.78~-2.44) |
| Saudi Arabia | 191.03 (140.2~256.92) | 239.99 (176.35~332.55) | 25.63 | 307.3 (228.8~408.77) | 109.23 (80.63~151.21) | -3.68 (-3.73~-3.63) |
| Senegal | 137.54 (103.73~184.86) | 183.49 (138.65~250.39) | 33.41 | 411.79 (308.69~553.99) | 251.06 (187.98~342.3) | -1.47 (-1.59~-1.35) |
| Serbia | 75.43 (54.13~102.65) | 45.71 (33.35~62.22) | -39.4 | 173.46 (124.06~235.01) | 120.33 (87.24~162.23) | -1.33 (-1.7~-0.95) |
| Seychelles | 0.47 (0.35~0.64) | 0.49 (0.37~0.67) | 4.26 | 121.59 (90.95~163.14) | 104.04 (77.14~141.36) | -0.16 (-0.43~0.12) |
| Sierra Leone | 63.05 (48.37~85.21) | 104.46 (78.75~142.18) | 65.68 | 359.04 (277.07~484.14) | 241.83 (183.44~321.7) | -1.4 (-1.55~-1.24) |
| Singapore | 15.26 (10.95~21.35) | 17.96 (12.73~25.88) | 17.69 | 74.65 (53.78~104.21) | 53 (37.72~74.38) | -1.34 (-1.78~-0.89) |
| Slovakia | 55.36 (40.11~73.83) | 42.63 (31.44~57.11) | -22.99 | 220.3 (158.26~294.67) | 170.71 (126.28~226.21) | -0.42 (-1.08~0.25) |
| Slovenia | 12.16 (8.9~16.4) | 11.95 (8.37~16.68) | -1.73 | 123.38 (90.7~167.07) | 141.76 (100.09~196.13) | 1.18 (0.9~1.46) |
| Solomon Islands | 8.5 (6.39~11.23) | 12.78 (9.45~17.31) | 50.35 | 615.5 (455.35~809.18) | 393.91 (289.65~534.16) | -1.58 (-1.67~-1.49) |
| Somalia | 139.27 (107.13~185.37) | 352.51 (263.92~468.28) | 153.11 | 462.72 (355.09~612.37) | 403.13 (302.3~532.44) | -0.49 (-0.54~-0.44) |
| South Africa | 145.39 (110.88~188.79) | 127.96 (98.36~169) | -11.99 | 71.73 (54.78~93.56) | 40.59 (31.34~53.1) | -2.16 (-2.32~-2) |
| South Korea | 212.43 (148.62~299.3) | 114.8 (98.77~133.59) | -45.96 | 75.21 (52.78~105.62) | 47.95 (41.11~55.93) | -2.27 (-2.6~-1.93) |
| South Sudan | 95.59 (72.13~126.95) | 143.5 (108.84~192.32) | 50.12 | 376.02 (287.11~490.52) | 333.72 (252.64~450.46) | -0.4 (-0.45~-0.34) |
| Spain | 297.5 (215.37~411.14) | 329.13 (236.86~459.82) | 10.63 | 153.87 (111.99~212.67) | 157.58 (114.02~214.03) | 0.48 (0.33~0.63) |
| Sri Lanka | 117.5 (86.21~158.61) | 107.04 (77.27~148.72) | -8.9 | 123.93 (91.14~167.71) | 96.66 (69.7~133.56) | -0.54 (-0.72~-0.36) |
| Sudan | 335.16 (254.9~458.33) | 379.84 (288.55~508.75) | 13.33 | 360 (270.32~489.97) | 176.67 (133.76~237.59) | -2.53 (-2.69~-2.36) |
| Suriname | 3.49 (2.55~4.75) | 3.78 (2.78~5.13) | 8.31 | 172.84 (126.68~233.69) | 131.58 (96.37~178.31) | -0.83 (-0.96~-0.7) |
| Sweden | 164.82 (121.67~219.19) | 150.37 (108.43~204.36) | -8.77 | 421.21 (310.54~558.6) | 331.43 (241.33~450.32) | -0.47 (-0.77~-0.17) |
| Switzerland | 77.07 (54.88~106.86) | 90 (64~122.66) | 16.78 | 210.93 (149.94~291.04) | 215.39 (153.85~292.13) | 0.11 (-0.07~0.3) |
| Syrian Arab Republic | 146.69 (111.65~193.01) | 72.75 (54.11~97.27) | -50.41 | 273.17 (205.61~361.55) | 102.69 (76.1~138.67) | -2.87 (-3.38~-2.35) |
| Taiwan | 240.97 (174.31~332.41) | 210.35 (150.09~288.9) | -12.71 | 196.75 (143.3~270.46) | 172.32 (123.61~237.45) | -0.84 (-1.31~-0.37) |
| Tajikistan | 127.67 (93.45~168.25) | 170.88 (124.39~231.84) | 33.85 | 501.28 (375.74~658.47) | 322.1 (237.28~434) | -1.31 (-1.55~-1.07) |
| Tanzania | 467.72 (354.43~622.73) | 789.41 (597.16~1061.39) | 68.78 | 395.59 (299.88~524.84) | 277.46 (211.21~373.4) | -1.09 (-1.18~-1.01) |
| Thailand | 313.88 (237.47~421.52) | 185.69 (137.96~250.37) | -40.84 | 93.26 (70.8~124.16) | 55.08 (40.75~74.08) | -1.62 (-1.74~-1.51) |
| Timor-Leste | 13.32 (9.92~17.75) | 13.41 (9.93~18.05) | 0.68 | 356.56 (267.59~473.14) | 225.01 (167.33~304.44) | -1.73 (-1.98~-1.48) |
| Togo | 65.08 (49.57~87.65) | 94.84 (71.08~129.27) | 45.73 | 392.66 (300.68~529.97) | 229.56 (171.92~311.03) | -1.62 (-1.76~-1.48) |
| Tokelau | 0.04 (0.03~0.05) | 0.02 (0.02~0.03) | -50 | 557.28 (413.24~738.2) | 379.01 (275.23~518.05) | -1.34 (-1.36~-1.32) |
| Tonga | 1.57 (1.16~2.14) | 1.49 (1.09~2.01) | -5.1 | 390.09 (287.09~526.98) | 313.29 (227.58~422.09) | -0.66 (-0.73~-0.59) |
| Trinidad and Tobago | 8.92 (6.52~12.23) | 6.74 (4.9~9.26) | -24.44 | 136.68 (100.11~184.88) | 95.62 (70.52~129.22) | -0.76 (-1.02~-0.51) |
| Tunisia | 75.96 (57.05~101.89) | 63.62 (45.88~88.01) | -16.25 | 184.78 (139.63~248.87) | 95.91 (69.62~131.68) | -1.92 (-2.11~-1.73) |
| Turkey | 757.05 (568.56~1013.46) | 674.16 (495.39~919.38) | -10.95 | 244.04 (182.12~322.53) | 149.19 (110~201.82) | -1.62 (-1.73~-1.5) |
| Turkmenistan | 81.44 (59.46~109.38) | 67.44 (48.41~90.77) | -17.19 | 430.19 (316.91~584.46) | 262.41 (188.74~353.13) | -1.42 (-1.84~-0.99) |
| Tuvalu | 0.18 (0.13~0.24) | 0.13 (0.09~0.17) | -27.78 | 366.3 (267.22~485.47) | 231.11 (166.98~317.05) | -1.62 (-1.65~-1.59) |
| Uganda | 343.3 (256.67~466.48) | 573.9 (426.69~779.31) | 67.17 | 442.56 (335.59~595.19) | 287.38 (217.29~389.03) | -1.58 (-1.76~-1.4) |
| UK | 619.22 (461.82~805.72) | 690.05 (511.23~923.97) | 11.44 | 216.34 (161.94~281.19) | 220.28 (164.55~291.73) | 0.43 (0.23~0.63) |
| Ukraine | 801.68 (596.34~1091.6) | 608.84 (449.41~813.56) | -24.05 | 330.24 (245.77~452.72) | 295.78 (219.25~394.48) | 0.75 (0.15~1.36) |
| United Arab Emirates | 15.57 (11.56~21.06) | 27.23 (18.97~38.04) | 74.89 | 211.8 (158.94~281.59) | 66.37 (48.57~89.29) | -3.73 (-3.84~-3.61) |
| United States Virgin Islands | 0.89 (0.66~1.19) | 0.44 (0.33~0.6) | -50.56 | 163.72 (121.77~221.16) | 102.78 (75.46~139.7) | -1.77 (-1.86~-1.69) |
| Uruguay | 60.37 (44.64~80.34) | 52.71 (39.35~72.32) | -12.69 | 401.04 (296.35~533.94) | 318.99 (237.44~434.63) | -0.93 (-1.08~-0.78) |
| USA | 1773.42 (1237.05~2451.61) | 1140.34 (970.71~1390.7) | -35.7 | 128.89 (90.49~178.25) | 76.28 (64.96~92.83) | -2.69 (-3.17~-2.21) |
| Uzbekistan | 426.84 (309.11~578.25) | 417.16 (302.27~560.71) | -2.27 | 391.34 (285.34~522.46) | 218.38 (158.65~291.91) | -1.77 (-2.15~-1.39) |
| Vanuatu | 3.25 (2.41~4.37) | 4.28 (3.15~5.66) | 31.69 | 467.5 (348.29~635.87) | 286.33 (211.4~380.44) | -1.8 (-1.84~-1.75) |
| Venezuela | 247.76 (186.15~337.01) | 210.13 (159.8~282.9) | -15.19 | 247.63 (186.69~333.47) | 143.24 (108.8~193.16) | -1.4 (-1.64~-1.16) |
| Vietnam | 514.73 (375.27~703.94) | 414.01 (303.97~572.35) | -19.57 | 139.5 (103.74~188.87) | 75.71 (56~104.12) | -1.74 (-1.89~-1.59) |
| Yemen | 230.62 (173.76~309.49) | 319.28 (242.29~421.52) | 38.44 | 422.14 (316.26~564.14) | 202.16 (153.73~267.61) | -2.64 (-2.83~-2.44) |
| Zambia | 146.24 (109.72~198.03) | 225.69 (169.47~307.61) | 54.33 | 406.32 (303.09~543.83) | 239.78 (180.53~325.97) | -1.78 (-1.94~-1.62) |
| Zimbabwe | 125.56 (94.84~169.86) | 144.7 (107.1~200.19) | 15.24 | 259.28 (195.48~352.53) | 175.33 (131.1~239.99) | -0.94 (-1.1~-0.79) |

ASIR, age-standardized incidence rate; CI, confidence interval; EAPC, estimated annual percentage change; UI, uncertainty interval.

Supplement Table S4 The death cases and age-standardized death rate of ectopic pregnancy in 1990 and 2019, and its temporal trends from 1990 to 2019.

| **Nation** | **Death cases No. 10^2^ (95% UI)** | | **Change in absolute  number (%)** | **ASDR per 100,000 No. 10^2^ (95% UI)** | | **1990-2019 EAPC  No. (95% CI)** |
| --- | --- | --- | --- | --- | --- | --- |
|  | **1990** | **2019** |  | **1990** | **2019** |  |
| Afghanistan | 0.05 (0.04~0.07) | 0.12 (0.08~0.17) | 140 | 0.12 (0.08~0.16) | 0.08 (0.05~0.11) | -1.27 (-1.71~-0.83) |
| Albania | 0 (0~0) | 0 (0~0) | NA | 0.02 (0.02~0.03) | 0.01 (0~0.01) | -1.94 (-2.43~-1.46) |
| Algeria | 0.07 (0.05~0.09) | 0.03 (0.02~0.04) | -57.14 | 0.06 (0.04~0.08) | 0.01 (0.01~0.02) | -4.99 (-5.2~-4.77) |
| American Samoa | 0 (0~0) | 0 (0~0) | NA | 0.07 (0.05~0.1) | 0.11 (0.07~0.16) | 1.37 (0.81~1.93) |
| Andorra | 0 (0~0) | 0 (0~0) | NA | 0 (0~0) | 0 (0~0) | -3.56 (-3.68~-3.44) |
| Angola | 0.47 (0.31~0.65) | 1.31 (0.82~1.82) | 178.72 | 1.08 (0.71~1.51) | 0.97 (0.6~1.37) | 0.05 (-0.22~0.33) |
| Antigua and Barbuda | 0 (0~0) | 0 (0~0) | NA | 0.03 (0.02~0.04) | 0.09 (0.07~0.12) | 4.94 (4.08~5.8) |
| Argentina | 0.01 (0.01~0.01) | 0.06 (0.05~0.07) | 500 | 0.01 (0.01~0.01) | 0.03 (0.02~0.03) | 4.62 (3.32~5.93) |
| Armenia | 0 (0~0) | 0 (0~0) | NA | 0.01 (0.01~0.01) | 0 (0~0) | -3.09 (-4.29~-1.88) |
| Australia | 0.01 (0.01~0.01) | 0 (0~0.01) | -100 | 0.01 (0.01~0.02) | 0 (0~0) | -4.15 (-5.03~-3.26) |
| Austria | 0.01 (0.01~0.01) | 0 (0~0) | -100 | 0.02 (0.01~0.02) | 0 (0~0) | -11.03 (-12.31~-9.73) |
| Azerbaijan | 0 (0~0) | 0 (0~0) | NA | 0.01 (0.01~0.01) | 0 (0~0) | -4.05 (-4.59~-3.5) |
| Bahamas | 0 (0~0) | 0 (0~0.01) | NA | 0.08 (0.06~0.1) | 0.19 (0.14~0.27) | 3.11 (2.17~4.05) |
| Bahrain | 0 (0~0) | 0 (0~0) | NA | 0.02 (0.02~0.03) | 0.01 (0.01~0.01) | -2.93 (-3.52~-2.34) |
| Bangladesh | 0.1 (0.07~0.12) | 0.31 (0.22~0.44) | 210 | 0.02 (0.01~0.02) | 0.03 (0.02~0.05) | 5.68 (4.21~7.18) |
| Barbados | 0 (0~0) | 0 (0~0) | NA | 0.05 (0.04~0.06) | 0.14 (0.11~0.19) | 4.59 (3.52~5.68) |
| Belarus | 0.01 (0.01~0.01) | 0 (0~0) | -100 | 0.02 (0.02~0.03) | 0.01 (0~0.01) | -4.37 (-4.82~-3.93) |
| Belgium | 0 (0~0) | 0 (0~0) | NA | 0.01 (0.01~0.01) | 0 (0~0) | -5.9 (-6.89~-4.89) |
| Belize | 0 (0~0) | 0 (0~0) | NA | 0.01 (0.01~0.02) | 0.06 (0.05~0.08) | 5.2 (3.83~6.58) |
| Benin | 0.41 (0.32~0.53) | 0.49 (0.31~0.74) | 19.51 | 2.09 (1.61~2.68) | 0.88 (0.57~1.33) | -2.92 (-3.3~-2.53) |
| Bermuda | 0 (0~0) | 0 (0~0) | NA | 0.01 (0.01~0.02) | 0 (0~0.01) | -3.55 (-3.96~-3.13) |
| Bhutan | 0 (0~0) | 0 (0~0) | NA | 0.07 (0.04~0.1) | 0.03 (0.02~0.04) | -3.68 (-3.99~-3.36) |
| Bolivia | 0.01 (0.01~0.02) | 0.11 (0.07~0.15) | 1000 | 0.05 (0.04~0.06) | 0.18 (0.11~0.25) | 4.14 (3.06~5.24) |
| Bosnia and Herzegovina | 0 (0~0) | 0 (0~0) | NA | 0.01 (0.01~0.01) | 0 (0~0) | -11.66 (-12.72~-10.59) |
| Botswana | 0.08 (0.05~0.11) | 0.06 (0.03~0.1) | -25 | 1.17 (0.75~1.67) | 0.44 (0.25~0.68) | -2.91 (-3.57~-2.25) |
| Brazil | 0.95 (0.8~1.11) | 0.45 (0.38~0.53) | -52.63 | 0.12 (0.1~0.14) | 0.04 (0.03~0.05) | -2.84 (-3.62~-2.05) |
| Brunei Darussalam | 0 (0~0) | 0 (0~0) | NA | 0.18 (0.13~0.25) | 0.08 (0.06~0.11) | -2.03 (-2.6~-1.45) |
| Bulgaria | 0.01 (0.01~0.02) | 0 (0~0) | -100 | 0.03 (0.03~0.04) | 0 (0~0.01) | -10.56 (-12.01~-9.09) |
| Burkina Faso | 0.49 (0.33~0.67) | 0.91 (0.6~1.31) | 85.71 | 1.33 (0.9~1.83) | 0.92 (0.6~1.34) | -1.63 (-1.94~-1.32) |
| Burundi | 0.34 (0.24~0.47) | 0.44 (0.32~0.6) | 29.41 | 1.55 (1.07~2.15) | 0.91 (0.64~1.27) | -2.32 (-2.74~-1.91) |
| Cambodia | 0.07 (0.05~0.09) | 0.07 (0.05~0.1) | 0 | 0.13 (0.09~0.18) | 0.08 (0.05~0.11) | -1.76 (-2.22~-1.31) |
| Cameroon | 0.79 (0.59~1.01) | 1.67 (1.01~2.42) | 111.39 | 1.78 (1.34~2.29) | 1.19 (0.73~1.74) | -1.01 (-1.4~-0.61) |
| Canada | 0.01 (0.01~0.01) | 0.01 (0~0.01) | 0 | 0.01 (0~0.01) | 0 (0~0) | -1.62 (-2.32~-0.92) |
| Cape Verde | 0 (0~0.01) | 0 (0~0) | NA | 0.3 (0.22~0.37) | 0.09 (0.06~0.12) | -4.06 (-4.7~-3.42) |
| Central African Republic | 0.15 (0.11~0.2) | 0.25 (0.16~0.36) | 66.67 | 1.25 (0.9~1.71) | 0.98 (0.64~1.43) | -0.37 (-0.73~-0.01) |
| Chad | 0.39 (0.29~0.52) | 1.29 (0.89~1.74) | 230.77 | 1.56 (1.14~2.07) | 1.92 (1.33~2.61) | 0.75 (0.49~1.01) |
| Chile | 0.03 (0.03~0.04) | 0.01 (0~0.01) | -66.67 | 0.05 (0.04~0.06) | 0.01 (0~0.01) | -6.72 (-7.54~-5.91) |
| China | 3.87 (3.04~4.81) | 0.61 (0.46~0.78) | -84.24 | 0.06 (0.04~0.07) | 0.01 (0.01~0.01) | -6.38 (-6.92~-5.83) |
| Colombia | 0.13 (0.11~0.16) | 0.09 (0.06~0.13) | -30.77 | 0.07 (0.06~0.08) | 0.04 (0.03~0.05) | -2.32 (-2.94~-1.69) |
| Comoros | 0.01 (0~0.02) | 0.01 (0.01~0.02) | 0 | 0.82 (0.21~1.3) | 0.36 (0.19~0.55) | -3.51 (-4.06~-2.96) |
| Cook Islands | 0 (0~0) | 0 (0~0) | NA | 0.01 (0.01~0.02) | 0 (0~0.01) | -3.54 (-3.92~-3.17) |
| Costa Rica | 0 (0~0) | 0 (0~0) | NA | 0.01 (0.01~0.02) | 0.01 (0~0.01) | -4.88 (-5.9~-3.84) |
| Croatia | 0 (0~0) | 0 (0~0) | NA | 0.01 (0.01~0.02) | 0 (0~0) | -9.37 (-10.47~-8.25) |
| Cuba | 0.01 (0.01~0.01) | 0.03 (0.02~0.04) | 200 | 0.01 (0.01~0.01) | 0.06 (0.05~0.08) | 7.38 (5.72~9.05) |
| Cyprus | 0 (0~0) | 0 (0~0) | NA | 0.01 (0.01~0.02) | 0 (0~0) | -8.71 (-11.65~-5.66) |
| Czech Republic | 0.01 (0.01~0.01) | 0 (0~0) | -100 | 0.02 (0.02~0.03) | 0 (0~0) | -10.51 (-11.83~-9.17) |
| Democratic Republic of the Congo | 2.06 (1.42~2.81) | 4.58 (3.28~5.99) | 122.33 | 1.31 (0.9~1.77) | 1.2 (0.86~1.58) | 0.61 (0.24~0.98) |
| Denmark | 0 (0~0) | 0 (0~0) | NA | 0 (0~0) | 0 (0~0) | -4.54 (-6.16~-2.88) |
| Djibouti | 0.02 (0.01~0.03) | 0.06 (0.03~0.1) | 200 | 1.14 (0.74~1.63) | 0.97 (0.5~1.55) | -0.6 (-0.8~-0.4) |
| Dominica | 0 (0~0) | 0 (0~0) | NA | 0.06 (0.04~0.08) | 0.42 (0.29~0.6) | 7.07 (5.3~8.86) |
| Dominican Republic | 0.01 (0.01~0.01) | 0.04 (0.02~0.05) | 300 | 0.03 (0.02~0.04) | 0.06 (0.04~0.09) | 3.43 (2.9~3.97) |
| Ecuador | 0.01 (0~0.01) | 0.06 (0.05~0.09) | 500 | 0.01 (0.01~0.01) | 0.07 (0.05~0.1) | 6.83 (5.35~8.34) |
| Egypt | 0.09 (0.07~0.11) | 0.06 (0.04~0.09) | -33.33 | 0.03 (0.03~0.04) | 0.01 (0.01~0.02) | -3.03 (-3.22~-2.84) |
| El Salvador | 0.02 (0.02~0.03) | 0.02 (0.01~0.03) | 0 | 0.08 (0.06~0.1) | 0.05 (0.03~0.07) | -1.31 (-1.95~-0.66) |
| Equatorial Guinea | 0.02 (0.02~0.03) | 0.03 (0.02~0.05) | 50 | 1.28 (0.85~1.79) | 0.48 (0.26~0.84) | -3.14 (-3.62~-2.66) |
| Eritrea | 0.14 (0.09~0.2) | 0.35 (0.23~0.52) | 150 | 1.1 (0.71~1.56) | 1.09 (0.72~1.64) | -0.16 (-0.74~0.42) |
| Estonia | 0 (0~0.01) | 0 (0~0) | NA | 0.06 (0.05~0.08) | 0 (0~0) | -16.2 (-18.35~-13.99) |
| Eswatini | 0.03 (0.02~0.04) | 0.02 (0.01~0.03) | -33.33 | 0.94 (0.64~1.29) | 0.25 (0.13~0.43) | -4.41 (-5.13~-3.7) |
| Ethiopia | 2.38 (1.73~3.21) | 2.83 (2.02~4) | 18.91 | 1.27 (0.92~1.71) | 0.64 (0.46~0.91) | -2.72 (-2.9~-2.54) |
| Fiji | 0.01 (0.01~0.02) | 0.01 (0~0.01) | 0 | 0.3 (0.21~0.42) | 0.16 (0.11~0.24) | -2.74 (-3.1~-2.38) |
| Finland | 0 (0~0) | 0 (0~0) | NA | 0.01 (0~0.01) | 0 (0~0) | -5.01 (-6.09~-3.91) |
| France | 0.03 (0.02~0.03) | 0.01 (0.01~0.01) | -66.67 | 0.01 (0.01~0.01) | 0 (0~0.01) | -3.02 (-3.58~-2.46) |
| Gabon | 0.05 (0.04~0.07) | 0.05 (0.03~0.07) | 0 | 1.31 (0.97~1.72) | 0.52 (0.32~0.79) | -3.34 (-3.87~-2.81) |
| Gambia | 0.08 (0.05~0.11) | 0.15 (0.1~0.21) | 87.5 | 1.95 (1.28~2.77) | 1.47 (0.98~2.1) | -1.08 (-1.5~-0.66) |
| Georgia | 0 (0~0) | 0 (0~0) | NA | 0.01 (0.01~0.01) | 0.01 (0.01~0.01) | 4.59 (3.04~6.16) |
| Germany | 0.02 (0.02~0.03) | 0.01 (0~0.01) | -50 | 0.01 (0~0.01) | 0 (0~0) | -5.55 (-6.08~-5.02) |
| Ghana | 0.69 (0.47~0.91) | 0.91 (0.59~1.24) | 31.88 | 1 (0.69~1.33) | 0.52 (0.34~0.7) | -1.57 (-1.9~-1.24) |
| Greece | 0 (0~0.01) | 0 (0~0) | NA | 0.01 (0.01~0.01) | 0 (0~0) | -3.4 (-4.64~-2.13) |
| Greenland | 0 (0~0) | 0 (0~0) | NA | 0.02 (0.01~0.03) | 0 (0~0.01) | -7.24 (-8.53~-5.92) |
| Grenada | 0 (0~0) | 0 (0~0) | NA | 0.05 (0.04~0.06) | 0.07 (0.06~0.09) | 2.6 (1.16~4.06) |
| Guam | 0 (0~0) | 0 (0~0) | NA | 0.01 (0~0.01) | 0.04 (0.03~0.05) | 7.97 (7.46~8.48) |
| Guatemala | 0.04 (0.03~0.05) | 0.05 (0.03~0.07) | 25 | 0.1 (0.07~0.13) | 0.05 (0.03~0.06) | -3.86 (-4.97~-2.74) |
| Guinea | 0.57 (0.42~0.74) | 0.87 (0.61~1.19) | 52.63 | 2.11 (1.56~2.75) | 1.48 (1.04~2.03) | -1.09 (-1.29~-0.88) |
| Guinea-Bissau | 0.08 (0.06~0.11) | 0.07 (0.05~0.11) | -12.5 | 1.77 (1.22~2.42) | 0.76 (0.51~1.07) | -2.8 (-3.09~-2.51) |
| Guyana | 0.01 (0~0.01) | 0.02 (0.01~0.03) | 100 | 0.14 (0.1~0.18) | 0.5 (0.33~0.71) | 4.64 (3.32~5.98) |
| Haiti | 0.04 (0.03~0.06) | 0.37 (0.24~0.52) | 825 | 0.15 (0.1~0.21) | 0.52 (0.34~0.73) | 5.51 (4.47~6.55) |
| Honduras | 0.06 (0.05~0.08) | 0.02 (0.01~0.03) | -66.67 | 0.31 (0.23~0.4) | 0.04 (0.02~0.06) | -7.32 (-7.64~-6.99) |
| Hungary | 0.02 (0.02~0.03) | 0 (0~0) | -100 | 0.04 (0.03~0.05) | 0 (0~0) | -9.22 (-10.68~-7.74) |
| Iceland | 0 (0~0) | 0 (0~0) | NA | 0 (0~0) | 0 (0~0) | -3.94 (-4.37~-3.51) |
| India | 18.31 (14.74~22.63) | 13.01 (9.6~17) | -28.95 | 0.46 (0.37~0.56) | 0.18 (0.13~0.23) | -3.66 (-3.95~-3.36) |
| Indonesia | 1.84 (1.44~2.36) | 0.77 (0.59~1.04) | -58.15 | 0.2 (0.16~0.26) | 0.05 (0.04~0.07) | -4.92 (-5.46~-4.39) |
| Iran | 0.18 (0.15~0.22) | 0.05 (0.04~0.06) | -72.22 | 0.07 (0.06~0.09) | 0.01 (0.01~0.01) | -6.93 (-7.38~-6.48) |
| Iraq | 0.09 (0.06~0.13) | 0.08 (0.05~0.12) | -11.11 | 0.13 (0.09~0.18) | 0.04 (0.03~0.06) | -4.44 (-4.94~-3.93) |
| Ireland | 0 (0~0) | 0 (0~0) | NA | 0.01 (0.01~0.01) | 0 (0~0) | -2.1 (-3.88~-0.3) |
| Israel | 0 (0~0) | 0 (0~0) | NA | 0.02 (0.01~0.02) | 0 (0~0) | -5.51 (-6.67~-4.34) |
| Italy | 0.03 (0.03~0.04) | 0 (0~0.01) | -100 | 0.01 (0.01~0.01) | 0 (0~0) | -5.88 (-6.35~-5.4) |
| Jamaica | 0 (0~0) | 0.01 (0.01~0.01) | Inf | 0.01 (0.01~0.01) | 0.06 (0.04~0.08) | 6.61 (4.06~9.22) |
| Japan | 0.08 (0.06~0.09) | 0.01 (0.01~0.01) | -87.5 | 0.01 (0.01~0.01) | 0 (0~0) | -6.38 (-6.66~-6.09) |
| Jordan | 0.01 (0.01~0.01) | 0 (0~0.01) | -100 | 0.05 (0.03~0.06) | 0.01 (0~0.01) | -8.3 (-8.91~-7.69) |
| Kazakhstan | 0.01 (0.01~0.01) | 0 (0~0.01) | -100 | 0.01 (0.01~0.01) | 0 (0~0.01) | -4.4 (-5.48~-3.3) |
| Kenya | 0.72 (0.54~0.94) | 1.23 (0.84~1.71) | 70.83 | 0.86 (0.64~1.12) | 0.52 (0.36~0.73) | -1.45 (-1.98~-0.92) |
| Kiribati | 0 (0~0) | 0.01 (0.01~0.01) | Inf | 0.87 (0.61~1.2) | 1.42 (0.98~1.96) | 1.85 (1.1~2.61) |
| Kuwait | 0 (0~0) | 0 (0~0) | NA | 0.01 (0~0.01) | 0 (0~0) | -4.89 (-6.38~-3.37) |
| Kyrgyzstan | 0 (0~0.01) | 0 (0~0) | NA | 0.02 (0.02~0.03) | 0.01 (0.01~0.01) | -1.13 (-1.79~-0.47) |
| Laos | 0.02 (0.01~0.03) | 0.01 (0.01~0.02) | -50 | 0.09 (0.06~0.13) | 0.04 (0.02~0.05) | -3.51 (-3.89~-3.13) |
| Latvia | 0 (0~0) | 0 (0~0) | NA | 0.01 (0.01~0.02) | 0 (0~0.01) | -4.82 (-6.34~-3.27) |
| Lebanon | 0.01 (0.01~0.01) | 0 (0~0) | -100 | 0.05 (0.04~0.07) | 0.01 (0.01~0.02) | -5.35 (-5.62~-5.08) |
| Lesotho | 0.08 (0.05~0.11) | 0.1 (0.06~0.15) | 25 | 1.01 (0.68~1.41) | 0.88 (0.5~1.37) | 1.36 (0.55~2.18) |
| Liberia | 0.16 (0.12~0.22) | 0.29 (0.2~0.4) | 81.25 | 2.06 (1.51~2.74) | 1.25 (0.85~1.72) | -1.25 (-1.57~-0.92) |
| Libya | 0.01 (0~0.01) | 0 (0~0.01) | -100 | 0.05 (0.03~0.06) | 0.01 (0.01~0.01) | -4.48 (-5.21~-3.75) |
| Lithuania | 0 (0~0.01) | 0 (0~0) | NA | 0.02 (0.02~0.03) | 0 (0~0) | -8.48 (-10.06~-6.87) |
| Luxembourg | 0 (0~0) | 0 (0~0) | NA | 0.01 (0.01~0.01) | 0 (0~0) | -3.87 (-4.17~-3.56) |
| Madagascar | 0.81 (0.63~1.01) | 1.33 (0.96~1.8) | 64.2 | 1.66 (1.3~2.1) | 1.1 (0.78~1.52) | -1.77 (-2.17~-1.37) |
| Malawi | 0.62 (0.45~0.81) | 0.57 (0.36~0.83) | -8.06 | 1.62 (1.18~2.13) | 0.69 (0.44~1.02) | -2.78 (-3.2~-2.36) |
| Malaysia | 0.1 (0.07~0.13) | 0.08 (0.05~0.11) | -20 | 0.11 (0.08~0.14) | 0.05 (0.03~0.07) | -3.18 (-3.61~-2.74) |
| Maldives | 0 (0~0) | 0 (0~0) | NA | 0.1 (0.08~0.14) | 0.01 (0.01~0.02) | -6.74 (-7.39~-6.1) |
| Mali | 0.53 (0.42~0.69) | 0.84 (0.56~1.19) | 58.49 | 1.42 (1.1~1.83) | 0.87 (0.57~1.22) | -2.19 (-2.47~-1.9) |
| Malta | 0 (0~0) | 0 (0~0) | NA | 0.02 (0.02~0.03) | 0.01 (0.01~0.01) | -2.46 (-2.8~-2.11) |
| Marshall Islands | 0 (0~0) | 0 (0~0) | NA | 0.27 (0.19~0.36) | 0.2 (0.11~0.3) | -1.36 (-1.65~-1.07) |
| Mauritania | 0.29 (0.22~0.37) | 0.35 (0.22~0.52) | 20.69 | 3.46 (2.69~4.49) | 1.93 (1.21~2.83) | -1.47 (-1.69~-1.24) |
| Mauritius | 0 (0~0) | 0 (0~0) | NA | 0.03 (0.02~0.03) | 0.03 (0.02~0.04) | 1.26 (-0.18~2.71) |
| Mexico | 0.41 (0.35~0.49) | 0.22 (0.16~0.29) | -46.34 | 0.09 (0.08~0.11) | 0.03 (0.02~0.04) | -3.86 (-4.32~-3.4) |
| Micronesia | 0 (0~0) | 0 (0~0) | NA | 0.35 (0.23~0.51) | 0.17 (0.06~0.28) | -2.85 (-3~-2.7) |
| Moldova | 0 (0~0) | 0 (0~0) | NA | 0.01 (0.01~0.01) | 0 (0~0) | -4.02 (-4.69~-3.35) |
| Monaco | 0 (0~0) | 0 (0~0) | NA | 0 (0~0) | 0 (0~0) | -3.3 (-3.65~-2.94) |
| Mongolia | 0.01 (0~0.01) | 0 (0~0.01) | -100 | 0.05 (0.04~0.07) | 0.02 (0.01~0.03) | -3.4 (-3.87~-2.93) |
| Montenegro | 0 (0~0) | 0 (0~0) | NA | 0 (0~0) | 0 (0~0) | -1.19 (-1.78~-0.59) |
| Morocco | 0.1 (0.07~0.13) | 0.05 (0.04~0.08) | -50 | 0.08 (0.06~0.1) | 0.03 (0.02~0.04) | -3.77 (-4.23~-3.32) |
| Mozambique | 0.48 (0.34~0.65) | 1.07 (0.65~1.55) | 122.92 | 0.86 (0.61~1.17) | 0.84 (0.5~1.21) | 1.19 (0.64~1.74) |
| Myanmar | 0.19 (0.13~0.29) | 0.43 (0.28~0.63) | 126.32 | 0.09 (0.06~0.13) | 0.14 (0.09~0.21) | 1.61 (1.27~1.94) |
| Namibia | 0.05 (0.03~0.07) | 0.05 (0.03~0.09) | 0 | 0.87 (0.53~1.2) | 0.42 (0.23~0.69) | -2.17 (-2.52~-1.81) |
| Nauru | 0 (0~0) | 0 (0~0) | NA | 0.43 (0.29~0.64) | 0.23 (0.14~0.37) | -2.43 (-2.79~-2.06) |
| Nepal | 0.07 (0.05~0.09) | 0.02 (0.01~0.03) | -71.43 | 0.07 (0.05~0.09) | 0.01 (0.01~0.02) | -6.32 (-6.88~-5.75) |
| Netherlands | 0.01 (0.01~0.01) | 0 (0~0) | -100 | 0.01 (0.01~0.01) | 0 (0~0) | -7.3 (-8.04~-6.56) |
| New Zealand | 0 (0~0) | 0 (0~0) | NA | 0.01 (0~0.01) | 0 (0~0) | -1.81 (-2.55~-1.06) |
| Nicaragua | 0 (0~0) | 0 (0~0) | NA | 0.01 (0.01~0.01) | 0.01 (0~0.01) | -1.6 (-2.53~-0.65) |
| Niger | 0.44 (0.31~0.57) | 1.18 (0.75~1.66) | 168.18 | 1.37 (0.97~1.82) | 1.35 (0.87~1.91) | -0.34 (-0.58~-0.1) |
| Nigeria | 4.16 (2.59~6.46) | 7.1 (4.3~11.7) | 70.67 | 1.1 (0.69~1.71) | 0.69 (0.42~1.14) | -1.79 (-2.32~-1.25) |
| Niue | 0 (0~0) | 0 (0~0) | NA | 0.18 (0.12~0.28) | 0.08 (0.04~0.13) | -3.35 (-3.55~-3.15) |
| North Korea | 0.25 (0.15~0.39) | 0.03 (0.02~0.05) | -88 | 0.22 (0.13~0.34) | 0.02 (0.01~0.04) | -8.11 (-8.38~-7.83) |
| North Macedonia | 0 (0~0) | 0 (0~0) | NA | 0.01 (0.01~0.02) | 0 (0~0) | -6.41 (-6.96~-5.86) |
| Northern Mariana Islands | 0 (0~0) | 0 (0~0) | NA | 0.15 (0.1~0.21) | 0.15 (0.1~0.21) | 0.03 (-0.69~0.75) |
| Norway | 0 (0~0) | 0 (0~0) | NA | 0.01 (0.01~0.01) | 0 (0~0) | -7.45 (-9.2~-5.67) |
| Oman | 0 (0~0.01) | 0 (0~0) | NA | 0.06 (0.04~0.08) | 0.01 (0.01~0.01) | -6.45 (-6.82~-6.07) |
| Pakistan | 1.92 (1.48~2.46) | 4.44 (3.09~6.01) | 131.25 | 0.41 (0.31~0.52) | 0.39 (0.27~0.52) | -0.36 (-0.8~0.08) |
| Palau | 0 (0~0) | 0 (0~0) | NA | 0.04 (0.02~0.05) | 0.02 (0.01~0.03) | -1.97 (-2.05~-1.9) |
| Palestine | 0 (0~0.01) | 0 (0~0) | NA | 0.06 (0.04~0.08) | 0.01 (0.01~0.01) | -5.58 (-5.98~-5.19) |
| Panama | 0.01 (0.01~0.01) | 0.01 (0.01~0.02) | 0 | 0.05 (0.04~0.06) | 0.07 (0.05~0.1) | 1.89 (1.4~2.38) |
| Papua New Guinea | 0.04 (0.02~0.05) | 0.09 (0.06~0.12) | 125 | 0.2 (0.14~0.27) | 0.17 (0.12~0.25) | -0.54 (-0.84~-0.23) |
| Paraguay | 0 (0~0) | 0.01 (0.01~0.02) | Inf | 0.01 (0.01~0.01) | 0.03 (0.02~0.05) | 3.62 (1.88~5.39) |
| Peru | 0.06 (0.05~0.07) | 0.11 (0.07~0.16) | 83.33 | 0.05 (0.04~0.07) | 0.06 (0.04~0.09) | 0.13 (-1.05~1.32) |
| Philippines | 0.6 (0.48~0.74) | 0.65 (0.45~0.92) | 8.33 | 0.19 (0.15~0.23) | 0.11 (0.08~0.16) | -1.52 (-1.64~-1.39) |
| Poland | 0.05 (0.04~0.06) | 0 (0~0) | -100 | 0.03 (0.02~0.03) | 0 (0~0) | -11.06 (-12.29~-9.81) |
| Portugal | 0.01 (0.01~0.01) | 0 (0~0) | -100 | 0.02 (0.01~0.02) | 0 (0~0) | -8.95 (-10.17~-7.71) |
| Puerto Rico | 0 (0~0.01) | 0 (0~0) | NA | 0.02 (0.02~0.03) | 0.01 (0.01~0.01) | -4.49 (-5.54~-3.43) |
| Qatar | 0 (0~0) | 0 (0~0) | NA | 0.05 (0.03~0.07) | 0.01 (0~0.01) | -6.86 (-7.29~-6.44) |
| Republic of Congo | 0.13 (0.09~0.17) | 0.22 (0.14~0.31) | 69.23 | 1.22 (0.87~1.69) | 0.8 (0.51~1.17) | -0.7 (-1.35~-0.04) |
| Romania | 0 (0~0.01) | 0 (0~0.01) | NA | 0 (0~0.01) | 0.01 (0~0.01) | 0.49 (-0.6~1.6) |
| Russia | 1.07 (0.91~1.25) | 0.08 (0.05~0.1) | -92.52 | 0.13 (0.11~0.16) | 0.01 (0.01~0.01) | -9.79 (-10.27~-9.3) |
| Rwanda | 0.18 (0.14~0.25) | 0.16 (0.1~0.23) | -11.11 | 0.66 (0.49~0.91) | 0.26 (0.17~0.38) | -3.35 (-3.75~-2.95) |
| Saint Helena | 0.79 (0.55~1.08) | 1.5 (0.96~2.19) | 89.87 | 1.6 (1.12~2.19) | 1.23 (0.77~1.8) | -0.56 (-0.89~-0.23) |
| Saint Kitts and Nevis | 0 (0~0) | 0 (0~0) | NA | 0.02 (0.01~0.02) | 0.02 (0.01~0.04) | 1.83 (1.1~2.55) |
| Saint Lucia | 0 (0~0) | 0 (0~0) | NA | 0.01 (0.01~0.01) | 0.01 (0.01~0.02) | 1.75 (1.21~2.29) |
| Saint Vincent | 0 (0~0) | 0 (0~0) | NA | 0.18 (0.14~0.21) | 0.24 (0.19~0.31) | 1.3 (0.98~1.62) |
| Samoa | 0 (0~0) | 0 (0~0) | NA | 0.1 (0.06~0.15) | 0.04 (0.02~0.06) | -4.2 (-4.7~-3.69) |
| San Marino | 0 (0~0) | 0 (0~0) | NA | 0 (0~0) | 0 (0~0) | -1.37 (-1.49~-1.25) |
| Sao Tome and Principe | 0 (0~0.01) | 0 (0~0) | NA | 0.79 (0.42~1.16) | 0.31 (0.21~0.45) | -4.11 (-4.84~-3.37) |
| Saudi Arabia | 0.03 (0.02~0.04) | 0.05 (0.03~0.07) | 66.67 | 0.05 (0.03~0.07) | 0.02 (0.01~0.03) | -2.05 (-2.51~-1.59) |
| Senegal | 0.54 (0.4~0.71) | 1.08 (0.72~1.5) | 100 | 1.72 (1.26~2.26) | 1.55 (1.03~2.16) | 0.22 (-0.04~0.48) |
| Serbia | 0 (0~0) | 0 (0~0) | NA | 0.01 (0~0.01) | 0 (0~0) | -3.71 (-4.09~-3.34) |
| Seychelles | 0 (0~0) | 0 (0~0) | NA | 0.12 (0.09~0.15) | 0.06 (0.04~0.08) | -1.84 (-2.29~-1.39) |
| Sierra Leone | 0.21 (0.14~0.29) | 0.61 (0.41~0.84) | 190.48 | 1.29 (0.88~1.79) | 1.5 (1.01~2.05) | 1.09 (0.74~1.44) |
| Singapore | 0 (0~0.01) | 0 (0~0) | NA | 0.02 (0.02~0.03) | 0 (0~0) | -8.11 (-9.38~-6.83) |
| Slovakia | 0 (0~0) | 0 (0~0) | NA | 0.01 (0.01~0.01) | 0 (0~0) | -4.14 (-5.25~-3.01) |
| Slovenia | 0 (0~0) | 0 (0~0) | NA | 0.02 (0.02~0.03) | 0 (0~0) | -6.82 (-8.84~-4.76) |
| Solomon Islands | 0.01 (0.01~0.01) | 0.02 (0.01~0.03) | 100 | 0.62 (0.4~0.94) | 0.61 (0.38~0.88) | -0.1 (-0.35~0.15) |
| Somalia | 0.32 (0.19~0.49) | 0.85 (0.55~1.25) | 165.62 | 1.16 (0.68~1.77) | 1.09 (0.69~1.61) | 0.03 (-0.14~0.21) |
| South Africa | 1.85 (1.49~2.26) | 0.71 (0.43~1.07) | -61.62 | 0.95 (0.77~1.16) | 0.22 (0.14~0.33) | -4.13 (-5.56~-2.68) |
| South Korea | 0.02 (0.02~0.02) | 0.01 (0~0.01) | -50 | 0.01 (0.01~0.01) | 0 (0~0) | -4.86 (-5.47~-4.25) |
| South Sudan | 0.14 (0.09~0.21) | 0.2 (0.11~0.34) | 42.86 | 0.65 (0.4~0.97) | 0.5 (0.28~0.86) | -0.5 (-0.77~-0.23) |
| Spain | 0.01 (0.01~0.01) | 0 (0~0) | -100 | 0 (0~0.01) | 0 (0~0) | -3.9 (-4.67~-3.12) |
| Sri Lanka | 0.28 (0.22~0.36) | 0.21 (0.14~0.3) | -25 | 0.29 (0.23~0.38) | 0.19 (0.13~0.28) | -1.42 (-1.6~-1.23) |
| Sudan | 0.07 (0.05~0.09) | 0.06 (0.04~0.1) | -14.29 | 0.07 (0.05~0.1) | 0.03 (0.02~0.05) | -2.55 (-2.93~-2.18) |
| Suriname | 0 (0~0.01) | 0.01 (0.01~0.01) | Inf | 0.22 (0.14~0.29) | 0.32 (0.22~0.43) | 1.63 (1.2~2.06) |
| Sweden | 0 (0~0) | 0 (0~0) | NA | 0 (0~0) | 0 (0~0) | -4.16 (-5.31~-2.99) |
| Switzerland | 0 (0~0) | 0 (0~0) | NA | 0 (0~0) | 0 (0~0) | -4.07 (-5.2~-2.93) |
| Syrian Arab Republic | 0.02 (0.01~0.03) | 0 (0~0.01) | -100 | 0.04 (0.03~0.06) | 0.01 (0~0.01) | -7.21 (-7.78~-6.63) |
| Taiwan | 0.03 (0.02~0.03) | 0.01 (0~0.01) | -66.67 | 0.02 (0.02~0.03) | 0 (0~0.01) | -5.7 (-6.43~-4.96) |
| Tajikistan | 0.01 (0.01~0.02) | 0 (0~0.01) | -100 | 0.05 (0.04~0.07) | 0.01 (0.01~0.01) | -7.36 (-8.13~-6.58) |
| Tanzania | 1.68 (1.21~2.25) | 2.5 (1.68~3.48) | 48.81 | 1.73 (1.23~2.34) | 1.01 (0.67~1.43) | -1.09 (-1.48~-0.71) |
| Thailand | 0.07 (0.05~0.1) | 0.07 (0.04~0.1) | 0 | 0.02 (0.01~0.03) | 0.02 (0.01~0.03) | -0.31 (-1.25~0.64) |
| Timor-Leste | 0.01 (0.01~0.01) | 0.01 (0~0.02) | 0 | 0.27 (0.16~0.38) | 0.24 (0.05~0.36) | -1.08 (-1.73~-0.43) |
| Togo | 0.24 (0.18~0.31) | 0.25 (0.16~0.36) | 4.17 | 1.55 (1.14~2.03) | 0.64 (0.41~0.91) | -3.2 (-3.66~-2.73) |
| Tokelau | 0 (0~0) | 0 (0~0) | NA | 0.28 (0.17~0.42) | 0.13 (0.08~0.21) | -2.7 (-2.8~-2.6) |
| Tonga | 0 (0~0) | 0 (0~0) | NA | 0.23 (0.17~0.3) | 0.14 (0.09~0.21) | -2.04 (-2.22~-1.85) |
| Trinidad and Tobago | 0 (0~0) | 0.01 (0~0.01) | Inf | 0.02 (0.02~0.03) | 0.08 (0.05~0.12) | 5.83 (4.37~7.31) |
| Tunisia | 0.01 (0.01~0.02) | 0.01 (0~0.01) | 0 | 0.03 (0.02~0.04) | 0.01 (0.01~0.01) | -4.15 (-4.3~-3.99) |
| Turkey | 0.11 (0.09~0.15) | 0.02 (0.01~0.03) | -81.82 | 0.04 (0.03~0.05) | 0 (0~0.01) | -8.93 (-9.97~-7.88) |
| Turkmenistan | 0.01 (0.01~0.01) | 0.01 (0.01~0.01) | 0 | 0.04 (0.03~0.05) | 0.04 (0.03~0.05) | -0.05 (-0.54~0.44) |
| Tuvalu | 0 (0~0) | 0 (0~0) | NA | 0.34 (0.23~0.48) | 0.14 (0.08~0.21) | -3.35 (-3.48~-3.22) |
| Uganda | 0.3 (0.19~0.44) | 0.98 (0.64~1.43) | 226.67 | 0.46 (0.28~0.67) | 0.56 (0.37~0.81) | 0.76 (0.12~1.41) |
| UK | 0.06 (0.05~0.07) | 0.01 (0.01~0.01) | -83.33 | 0.02 (0.02~0.02) | 0 (0~0) | -6.15 (-6.71~-5.59) |
| Ukraine | 0.02 (0.02~0.03) | 0.01 (0~0.01) | -50 | 0.01 (0.01~0.01) | 0 (0~0) | -4.14 (-4.97~-3.3) |
| United Arab Emirates | 0 (0~0) | 0 (0~0) | NA | 0.04 (0.02~0.05) | 0.01 (0~0.01) | -6.32 (-6.86~-5.78) |
| United States Virgin Islands | 0 (0~0) | 0 (0~0) | NA | 0.02 (0.01~0.02) | 0.01 (0.01~0.02) | -0.5 (-1.21~0.22) |
| Uruguay | 0 (0~0) | 0 (0~0) | NA | 0.02 (0.01~0.02) | 0 (0~0.01) | -5.65 (-6.49~-4.8) |
| USA | 0.41 (0.34~0.49) | 0.19 (0.16~0.23) | -53.66 | 0.03 (0.03~0.04) | 0.01 (0.01~0.02) | -2.35 (-2.67~-2.03) |
| Uzbekistan | 0.01 (0.01~0.01) | 0.01 (0.01~0.02) | 0 | 0.01 (0.01~0.01) | 0.01 (0.01~0.01) | -0.44 (-1.24~0.36) |
| Vanuatu | 0 (0~0) | 0 (0~0) | NA | 0.14 (0.08~0.22) | 0.12 (0.06~0.19) | -1.27 (-1.6~-0.95) |
| Venezuela | 0.01 (0.01~0.01) | 0.1 (0.07~0.14) | 900 | 0.01 (0.01~0.01) | 0.07 (0.05~0.1) | 6.44 (5.41~7.47) |
| Vietnam | 0.04 (0.03~0.07) | 0.02 (0.01~0.03) | -50 | 0.01 (0.01~0.02) | 0 (0~0) | -3.52 (-3.88~-3.17) |
| Yemen | 0.05 (0.03~0.08) | 0.15 (0.08~0.24) | 200 | 0.1 (0.05~0.15) | 0.1 (0.05~0.15) | -0.04 (-0.5~0.42) |
| Zambia | 0.33 (0.24~0.43) | 0.37 (0.24~0.53) | 12.12 | 1.13 (0.83~1.52) | 0.48 (0.3~0.7) | -3.31 (-3.58~-3.05) |
| Zimbabwe | 0.53 (0.4~0.68) | 0.74 (0.5~1.03) | 39.62 | 1.22 (0.91~1.58) | 0.95 (0.64~1.32) | 1.44 (0.29~2.6) |

ASDR, age-standardized incidence rate; CI, confidence interval; EAPC, estimated annual percentage change; UI, uncertainty interval.

Supplement Table S5 The DALY and age-standardized DALY rate of ectopic pregnancy in 1990 and 2019, and its temporal trends from 1990 to 2019.

| Nation | DALY cases No. 10^2^ (95% UI) | | Change in absolute  **number** (%) | Age Standardized DALY Rate per 100,000 No. 10^2^ (95% UI) | | 1990-2019 EAPC  No. (95% CI) |
| --- | --- | --- | --- | --- | --- | --- |
|  | 1990 | 2019 |  | 1990 | 2019 |  |
| Afghanistan | 3.17 (2.28~4.27) | 7.14 (5.09~10.32) | 125.24 | 6.68 (4.82~9.08) | 4.32 (3.07~6.13) | -1.28 (-1.71~-0.85) |
| Albania | 0.27 (0.21~0.34) | 0.08 (0.05~0.11) | -70.37 | 1.5 (1.17~1.87) | 0.6 (0.42~0.83) | -1.9 (-2.29~-1.51) |
| Algeria | 4.1 (3~5.58) | 2.19 (1.61~2.91) | -46.59 | 3.59 (2.6~4.92) | 0.93 (0.69~1.23) | -4.55 (-4.8~-4.3) |
| American Samoa | 0.01 (0.01~0.01) | 0.02 (0.01~0.02) | 100 | 4.23 (3.08~5.59) | 6.16 (4.3~8.61) | 1.09 (0.59~1.59) |
| Andorra | 0 (0~0) | 0 (0~0) | NA | 0.31 (0.21~0.42) | 0.2 (0.13~0.29) | -1.59 (-1.64~-1.54) |
| Angola | 26.94 (17.87~36.68) | 75.27 (47.79~103.89) | 179.4 | 60.19 (39.58~83.16) | 54.21 (33.99~75.71) | 0.09 (-0.2~0.37) |
| Antigua and Barbuda | 0.01 (0.01~0.01) | 0.03 (0.02~0.03) | 200 | 1.84 (1.48~2.31) | 5.54 (4.3~7.07) | 4.7 (3.9~5.51) |
| Argentina | 1.38 (0.96~1.95) | 4.35 (3.58~5.3) | 215.22 | 0.86 (0.59~1.22) | 1.86 (1.53~2.27) | 3.04 (2.18~3.91) |
| Armenia | 0.16 (0.13~0.2) | 0.05 (0.03~0.07) | -68.75 | 0.85 (0.66~1.07) | 0.34 (0.24~0.45) | -2.28 (-3.16~-1.38) |
| Australia | 0.72 (0.57~0.9) | 0.29 (0.23~0.37) | -59.72 | 0.8 (0.63~0.99) | 0.25 (0.2~0.31) | -3.81 (-4.62~-3) |
| Austria | 0.51 (0.4~0.64) | 0.17 (0.08~0.29) | -66.67 | 1.21 (0.96~1.54) | 0.42 (0.21~0.72) | -4.48 (-5.28~-3.68) |
| Azerbaijan | 0.32 (0.24~0.42) | 0.17 (0.11~0.23) | -46.88 | 0.74 (0.57~0.95) | 0.3 (0.21~0.42) | -3.03 (-3.47~-2.59) |
| Bahamas | 0.07 (0.06~0.09) | 0.24 (0.17~0.32) | 242.86 | 4.65 (3.74~5.82) | 11.5 (8.2~15.74) | 3.09 (2.17~4.03) |
| Bahrain | 0.04 (0.03~0.05) | 0.03 (0.03~0.05) | -25 | 1.5 (1.15~1.91) | 0.5 (0.37~0.68) | -2.93 (-3.47~-2.38) |
| Bangladesh | 7.36 (5.34~9.42) | 19.53 (14.01~26.33) | 165.35 | 1.34 (0.98~1.71) | 2.12 (1.52~2.86) | 4.38 (3.24~5.54) |
| Barbados | 0.04 (0.03~0.05) | 0.12 (0.09~0.15) | 200 | 2.96 (2.41~3.62) | 8.62 (6.4~11.41) | 4.47 (3.43~5.53) |
| Belarus | 0.8 (0.64~1) | 0.27 (0.17~0.41) | -66.25 | 1.51 (1.21~1.9) | 0.63 (0.4~0.95) | -2.71 (-3.2~-2.21) |
| Belgium | 0.31 (0.23~0.42) | 0.17 (0.1~0.28) | -45.16 | 0.6 (0.45~0.82) | 0.35 (0.21~0.55) | -2.84 (-3.41~-2.26) |
| Belize | 0.01 (0.01~0.01) | 0.09 (0.07~0.11) | 800 | 1.12 (0.87~1.42) | 3.82 (2.98~4.89) | 4.57 (3.37~5.79) |
| Benin | 23.38 (17.86~29.81) | 28 (17.48~42.15) | 19.76 | 112.64 (86.34~143.86) | 48.48 (30.74~72.45) | -2.86 (-3.24~-2.47) |
| Bermuda | 0 (0~0) | 0 (0~0) | NA | 0.84 (0.65~1.08) | 0.31 (0.23~0.42) | -2.97 (-3.3~-2.63) |
| Bhutan | 0.14 (0.08~0.2) | 0.08 (0.05~0.12) | -42.86 | 4.53 (2.64~6.39) | 1.75 (1.06~2.76) | -3.55 (-3.85~-3.26) |
| Bolivia | 1.02 (0.78~1.29) | 6.46 (4.14~9.05) | 533.33 | 3.27 (2.51~4.16) | 10.4 (6.67~14.63) | 3.73 (2.73~4.74) |
| Bosnia and Herzegovina | 0.18 (0.14~0.22) | 0.02 (0.01~0.03) | -88.89 | 0.73 (0.59~0.9) | 0.15 (0.09~0.22) | -7.31 (-8.23~-6.39) |
| Botswana | 4.72 (3.12~6.66) | 3.79 (2.14~5.8) | -19.7 | 69.29 (45.55~99.04) | 27.4 (15.55~42.02) | -2.77 (-3.41~-2.12) |
| Brazil | 55.91 (47.41~65.73) | 27.12 (22.95~32.25) | -51.49 | 7.04 (6~8.22) | 2.33 (1.98~2.77) | -2.67 (-3.41~-1.92) |
| Brunei Darussalam | 0.15 (0.11~0.22) | 0.12 (0.09~0.16) | -20 | 10.18 (7.38~14.33) | 4.72 (3.38~6.33) | -2.05 (-2.61~-1.49) |
| Bulgaria | 0.83 (0.69~1.01) | 0.1 (0.08~0.13) | -87.95 | 2.01 (1.66~2.44) | 0.38 (0.29~0.51) | -8.49 (-9.7~-7.25) |
| Burkina Faso | 26.72 (18.3~36.36) | 50.5 (33.31~73) | 89 | 69.73 (47.58~94.76) | 49.34 (32.84~71.63) | -1.49 (-1.76~-1.22) |
| Burundi | 18.88 (13.14~26.2) | 24.86 (17.71~33.66) | 31.67 | 81.98 (57.11~112.91) | 48.91 (34.89~67.08) | -2.24 (-2.66~-1.82) |
| Cambodia | 4.01 (2.83~5.66) | 4.14 (2.81~5.85) | 3.24 | 7.85 (5.55~10.96) | 4.52 (3.08~6.42) | -1.89 (-2.33~-1.45) |
| Cameroon | 45.14 (34.06~57.72) | 97.69 (58.93~141.32) | 116.42 | 98.18 (74.26~125.94) | 66.82 (40.65~96.68) | -0.93 (-1.32~-0.54) |
| Canada | 0.67 (0.51~0.84) | 0.51 (0.39~0.67) | -23.88 | 0.43 (0.33~0.53) | 0.31 (0.24~0.41) | -1.1 (-1.61~-0.58) |
| Cape Verde | 0.28 (0.21~0.36) | 0.16 (0.1~0.23) | -42.86 | 17.19 (13.03~21.76) | 5.12 (3.49~7.35) | -3.98 (-4.62~-3.34) |
| Central African Republic | 8.76 (6.37~11.73) | 14.17 (9.51~20.8) | 61.76 | 70.05 (50.25~94.81) | 54.86 (36.37~80.11) | -0.34 (-0.7~0.02) |
| Chad | 22.61 (16.74~30.06) | 76.36 (52.1~101.65) | 237.73 | 86.97 (63.99~115.57) | 108.11 (74.96~144.31) | 0.8 (0.54~1.06) |
| Chile | 2.24 (1.88~2.68) | 0.56 (0.4~0.78) | -75 | 3.05 (2.57~3.61) | 0.59 (0.43~0.84) | -5.36 (-5.9~-4.82) |
| China | 254.72 (203.84~314.19) | 48.19 (37.3~59.9) | -81.08 | 3.71 (2.97~4.58) | 0.69 (0.54~0.86) | -5.65 (-6.11~-5.19) |
| Colombia | 8.3 (6.72~10.06) | 5.96 (4.14~8.16) | -28.19 | 4.41 (3.61~5.29) | 2.36 (1.64~3.22) | -2.25 (-2.85~-1.66) |
| Comoros | 0.78 (0.18~1.26) | 0.68 (0.35~1.02) | -12.82 | 43.18 (10.65~68.28) | 19.22 (9.96~29.22) | -3.47 (-4.04~-2.9) |
| Cook Islands | 0 (0~0) | 0 (0~0) | NA | 0.98 (0.67~1.38) | 0.41 (0.24~0.59) | -2.73 (-2.98~-2.49) |
| Costa Rica | 0.16 (0.13~0.19) | 0.11 (0.08~0.15) | -31.25 | 0.95 (0.78~1.15) | 0.42 (0.32~0.56) | -4.39 (-5.24~-3.54) |
| Croatia | 0.22 (0.18~0.26) | 0.03 (0.02~0.04) | -86.36 | 0.88 (0.73~1.07) | 0.16 (0.1~0.24) | -5.24 (-6.07~-4.41) |
| Cuba | 0.45 (0.38~0.54) | 1.83 (1.35~2.42) | 306.67 | 0.71 (0.58~0.85) | 3.8 (2.82~5.03) | 6.96 (5.43~8.51) |
| Cyprus | 0.04 (0.02~0.05) | 0.01 (0.01~0.02) | -75 | 0.94 (0.63~1.36) | 0.15 (0.09~0.21) | -6.98 (-9.37~-4.52) |
| Czech Republic | 0.74 (0.62~0.88) | 0.1 (0.06~0.16) | -86.49 | 1.51 (1.24~1.8) | 0.24 (0.15~0.37) | -5.32 (-6.65~-3.96) |
| Democratic Republic of the Congo | 118.51 (82.17~159.43) | 261.11 (188.13~338.09) | 120.33 | 72.24 (49.96~98.26) | 65.97 (47.42~86.5) | 0.6 (0.22~0.97) |
| Denmark | 0.09 (0.06~0.13) | 0.08 (0.04~0.13) | -11.11 | 0.37 (0.25~0.51) | 0.32 (0.18~0.54) | -1.65 (-2.32~-0.97) |
| Djibouti | 1.25 (0.8~1.8) | 3.45 (1.8~5.57) | 176 | 62.6 (40.57~89.31) | 54.48 (28.37~87.26) | -0.52 (-0.74~-0.3) |
| Dominica | 0.01 (0.01~0.02) | 0.08 (0.06~0.12) | 700 | 3.81 (2.83~5.16) | 25.54 (17.31~36.07) | 7.03 (5.26~8.82) |
| Dominican Republic | 0.76 (0.6~0.96) | 2.25 (1.52~3.26) | 196.05 | 1.84 (1.46~2.32) | 3.81 (2.57~5.51) | 3.11 (2.59~3.63) |
| Ecuador | 0.5 (0.4~0.62) | 4.07 (2.94~5.66) | 714 | 0.93 (0.74~1.18) | 4.3 (3.11~5.99) | 5.85 (4.62~7.11) |
| Egypt | 6.01 (4.81~7.47) | 4.3 (3.02~6.12) | -28.45 | 2.2 (1.76~2.7) | 0.82 (0.58~1.18) | -2.91 (-3.08~-2.74) |
| El Salvador | 1.37 (1.1~1.67) | 1.01 (0.67~1.55) | -26.28 | 4.89 (3.99~5.94) | 2.83 (1.87~4.31) | -1.37 (-1.99~-0.74) |
| Equatorial Guinea | 1.36 (0.91~1.89) | 1.82 (0.97~3.11) | 33.82 | 71.17 (47.82~99.11) | 27.08 (14.49~46.95) | -3.08 (-3.57~-2.58) |
| Eritrea | 7.76 (5~11.09) | 19.76 (13.07~29.77) | 154.64 | 60.16 (38.91~85.57) | 60.07 (39.82~90.13) | -0.12 (-0.7~0.46) |
| Estonia | 0.29 (0.23~0.36) | 0.02 (0.01~0.04) | -93.1 | 3.92 (3.1~5) | 0.37 (0.2~0.63) | -8.78 (-10.14~-7.39) |
| Eswatini | 1.8 (1.25~2.4) | 0.86 (0.43~1.49) | -52.22 | 50.79 (35.12~68.97) | 13.93 (7.02~24.25) | -4.31 (-5.03~-3.59) |
| Ethiopia | 127.22 (93.26~170.37) | 155.39 (110.94~219.64) | 22.14 | 64.57 (47.34~86.71) | 33.41 (23.85~46.79) | -2.63 (-2.8~-2.46) |
| Fiji | 0.67 (0.47~0.95) | 0.43 (0.29~0.63) | -35.82 | 16.9 (12.02~23.84) | 9.2 (6.19~13.57) | -2.7 (-3.06~-2.35) |
| Finland | 0.14 (0.11~0.18) | 0.06 (0.04~0.09) | -57.14 | 0.56 (0.44~0.72) | 0.25 (0.17~0.37) | -2.62 (-3.1~-2.14) |
| France | 2.01 (1.61~2.52) | 1.18 (0.86~1.57) | -41.29 | 0.68 (0.54~0.85) | 0.43 (0.31~0.58) | -1.76 (-2.16~-1.37) |
| Gabon | 3.1 (2.33~4.03) | 2.84 (1.76~4.24) | -8.39 | 73.07 (54.45~96.1) | 29.25 (18.11~44.2) | -3.31 (-3.83~-2.79) |
| Gambia | 4.35 (2.91~6.14) | 8.33 (5.6~11.69) | 91.49 | 104.44 (69.77~147.03) | 78.29 (52.46~110.64) | -1.07 (-1.5~-0.64) |
| Georgia | 0.17 (0.13~0.21) | 0.12 (0.09~0.15) | -29.41 | 0.6 (0.46~0.75) | 0.73 (0.55~0.95) | 3.52 (2.43~4.62) |
| Germany | 1.63 (1.35~1.93) | 0.57 (0.43~0.73) | -65.03 | 0.39 (0.33~0.47) | 0.16 (0.12~0.21) | -3.43 (-3.82~-3.05) |
| Ghana | 40.11 (27.59~52.88) | 53.48 (34.42~73.07) | 33.33 | 56.62 (38.88~74.5) | 29.93 (19.48~40.58) | -1.48 (-1.79~-1.17) |
| Greece | 0.31 (0.25~0.36) | 0.16 (0.12~0.21) | -48.39 | 0.59 (0.49~0.7) | 0.36 (0.27~0.47) | -1.88 (-2.77~-0.98) |
| Greenland | 0 (0~0.01) | 0 (0~0) | NA | 1.39 (0.91~2.08) | 0.42 (0.29~0.59) | -6.29 (-7.45~-5.11) |
| Grenada | 0.01 (0.01~0.02) | 0.02 (0.02~0.03) | 100 | 3.13 (2.49~3.89) | 4.29 (3.41~5.41) | 2.51 (1.13~3.91) |
| Guam | 0 (0~0.01) | 0.02 (0.01~0.02) | Inf | 0.56 (0.39~0.75) | 2.24 (1.63~3.01) | 5.68 (5.26~6.1) |
| Guatemala | 2.37 (1.82~2.99) | 3.01 (2.15~4.13) | 27 | 6.35 (4.87~8.05) | 2.88 (2.06~3.95) | -3.81 (-4.86~-2.76) |
| Guinea | 32.03 (23.9~41.53) | 50.38 (35.47~68.57) | 57.29 | 115.82 (86.09~150.5) | 82.42 (58.23~111.78) | -1.03 (-1.23~-0.82) |
| Guinea-Bissau | 4.87 (3.39~6.63) | 4.39 (2.98~6.26) | -9.86 | 101.37 (70.88~138.05) | 43.17 (29.12~61.46) | -2.82 (-3.11~-2.52) |
| Guyana | 0.36 (0.26~0.47) | 1.28 (0.86~1.82) | 255.56 | 8.24 (6.06~10.72) | 29.49 (19.84~42.31) | 4.59 (3.29~5.91) |
| Haiti | 2.75 (1.92~3.86) | 21.19 (14.13~29.53) | 670.55 | 8.75 (6.12~12.29) | 29.77 (19.79~41.62) | 5.32 (4.32~6.33) |
| Honduras | 3.89 (2.99~4.95) | 1.3 (0.78~1.96) | -66.58 | 18.07 (13.87~23.14) | 2.43 (1.45~3.69) | -7.27 (-7.61~-6.92) |
| Hungary | 1.29 (1.06~1.55) | 0.1 (0.07~0.14) | -92.25 | 2.65 (2.16~3.2) | 0.25 (0.18~0.34) | -7.14 (-8.48~-5.78) |
| Iceland | 0 (0~0.01) | 0 (0~0.01) | NA | 0.34 (0.23~0.5) | 0.21 (0.13~0.34) | -1.3 (-1.52~-1.08) |
| India | 1082.19 (864.88~1344.86) | 766.11 (566.34~994.03) | -29.21 | 26.23 (21.19~32.54) | 10.22 (7.58~13.21) | -3.6 (-3.94~-3.26) |
| Indonesia | 105.31 (82.28~134.15) | 43.75 (33.15~58.21) | -58.46 | 11.25 (8.79~14.4) | 3.09 (2.34~4.11) | -4.85 (-5.35~-4.34) |
| Iran | 11.12 (8.92~13.47) | 3.24 (2.67~3.88) | -70.86 | 4.23 (3.4~5.14) | 0.66 (0.54~0.79) | -6.67 (-7.1~-6.23) |
| Iraq | 5.31 (3.69~7.33) | 4.85 (3.21~7.23) | -8.66 | 7.44 (5.15~10.3) | 2.24 (1.48~3.37) | -4.41 (-4.89~-3.92) |
| Ireland | 0.12 (0.09~0.16) | 0.1 (0.07~0.14) | -16.67 | 0.69 (0.53~0.89) | 0.41 (0.29~0.57) | -1.21 (-2.31~-0.1) |
| Israel | 0.29 (0.24~0.37) | 0.23 (0.14~0.36) | -20.69 | 1.18 (0.96~1.49) | 0.54 (0.33~0.84) | -2.97 (-3.69~-2.24) |
| Italy | 2.17 (1.82~2.59) | 0.59 (0.43~0.82) | -72.81 | 0.76 (0.64~0.9) | 0.25 (0.18~0.34) | -3.54 (-3.92~-3.17) |
| Jamaica | 0.08 (0.06~0.09) | 0.57 (0.38~0.81) | 612.5 | 0.57 (0.46~0.7) | 3.51 (2.35~5.01) | 5.9 (3.57~8.27) |
| Japan | 4.58 (3.84~5.42) | 0.83 (0.65~1.08) | -81.88 | 0.76 (0.63~0.91) | 0.18 (0.14~0.23) | -5.08 (-5.34~-4.83) |
| Jordan | 0.51 (0.38~0.69) | 0.34 (0.25~0.45) | -33.33 | 3.11 (2.28~4.08) | 0.58 (0.42~0.78) | -6.96 (-7.46~-6.45) |
| Kazakhstan | 0.81 (0.65~1) | 0.47 (0.35~0.63) | -41.98 | 0.93 (0.75~1.15) | 0.49 (0.36~0.65) | -2.62 (-3.21~-2.02) |
| Kenya | 40.52 (30.19~53.1) | 67.81 (46.52~93.91) | 67.35 | 45.11 (33.74~58.57) | 27.79 (19.11~38.59) | -1.43 (-1.96~-0.9) |
| Kiribati | 0.2 (0.14~0.28) | 0.52 (0.36~0.71) | 160 | 50.39 (35.5~69.99) | 81.09 (55.8~111.53) | 1.79 (1.05~2.55) |
| Kuwait | 0.04 (0.03~0.05) | 0.05 (0.04~0.07) | 25 | 0.42 (0.35~0.52) | 0.16 (0.12~0.21) | -4.61 (-5.86~-3.35) |
| Kyrgyzstan | 0.37 (0.3~0.45) | 0.3 (0.24~0.38) | -18.92 | 1.61 (1.3~1.93) | 0.85 (0.68~1.07) | -0.92 (-1.47~-0.37) |
| Laos | 1.05 (0.75~1.51) | 0.85 (0.56~1.15) | -19.05 | 5.37 (3.86~7.8) | 2.16 (1.43~2.93) | -3.52 (-3.88~-3.15) |
| Latvia | 0.15 (0.12~0.2) | 0.05 (0.03~0.07) | -66.67 | 1.22 (0.93~1.57) | 0.57 (0.34~0.89) | -2.47 (-3.35~-1.58) |
| Lebanon | 0.51 (0.35~0.7) | 0.21 (0.15~0.3) | -58.82 | 3.29 (2.24~4.53) | 0.77 (0.54~1.09) | -5 (-5.26~-4.74) |
| Lesotho | 4.44 (2.95~6.04) | 5.55 (3.18~8.69) | 25 | 55.4 (36.6~76.06) | 49.2 (28.07~75.91) | 1.43 (0.62~2.25) |
| Liberia | 9.14 (6.72~11.99) | 16.07 (11.02~21.89) | 75.82 | 110.58 (81.36~145.6) | 67.34 (45.89~92.43) | -1.21 (-1.54~-0.88) |
| Libya | 0.44 (0.31~0.61) | 0.25 (0.17~0.36) | -43.18 | 2.79 (1.97~3.83) | 0.61 (0.42~0.88) | -4.36 (-5.02~-3.69) |
| Lithuania | 0.31 (0.25~0.38) | 0.05 (0.03~0.08) | -83.87 | 1.67 (1.35~2.03) | 0.39 (0.23~0.63) | -4.57 (-5.65~-3.48) |
| Luxembourg | 0.02 (0.01~0.02) | 0.01 (0.01~0.02) | -50 | 0.73 (0.59~0.91) | 0.39 (0.28~0.52) | -2.42 (-2.64~-2.19) |
| Madagascar | 46.6 (36.35~58.06) | 74.06 (53.58~98.16) | 58.93 | 90.79 (71.07~113.5) | 58.79 (42.44~79.46) | -1.81 (-2.19~-1.42) |
| Malawi | 34.37 (25.3~45.36) | 32.7 (20.66~47.42) | -4.86 | 85.71 (62.9~112.4) | 37.64 (23.83~54.99) | -2.7 (-3.13~-2.26) |
| Malaysia | 5.79 (4.48~7.5) | 4.64 (3.17~6.66) | -19.86 | 6.24 (4.83~8) | 2.67 (1.83~3.84) | -3.19 (-3.61~-2.76) |
| Maldives | 0.06 (0.04~0.09) | 0.02 (0.02~0.03) | -66.67 | 6.24 (4.6~8.62) | 0.96 (0.71~1.26) | -6.41 (-7.05~-5.78) |
| Mali | 31.38 (24.71~40.36) | 50.16 (33.18~72) | 59.85 | 81.22 (63.28~104.47) | 49.65 (33~70.47) | -2.14 (-2.42~-1.87) |
| Malta | 0.03 (0.02~0.03) | 0.01 (0.01~0.01) | -66.67 | 1.37 (1.08~1.67) | 0.62 (0.49~0.76) | -2.27 (-2.61~-1.92) |
| Marshall Islands | 0.03 (0.02~0.04) | 0.03 (0.02~0.05) | 0 | 15.77 (11.15~20.79) | 11.34 (6.68~17.13) | -1.35 (-1.63~-1.06) |
| Mauritania | 15.79 (12.41~20.02) | 19.5 (12.23~29.25) | 23.5 | 183.16 (143.04~234.67) | 103.08 (65.1~152.99) | -1.42 (-1.65~-1.19) |
| Mauritius | 0.11 (0.09~0.13) | 0.12 (0.09~0.16) | 9.09 | 1.7 (1.37~2.08) | 1.92 (1.37~2.61) | 1.03 (-0.31~2.4) |
| Mexico | 25.77 (22.01~30.26) | 14.07 (10.75~18.19) | -45.4 | 5.66 (4.82~6.61) | 2.08 (1.59~2.69) | -3.68 (-4.12~-3.24) |
| Micronesia | 0.09 (0.06~0.14) | 0.05 (0.02~0.08) | -44.44 | 20.37 (13.56~29.59) | 10.18 (3.83~16.56) | -2.79 (-2.94~-2.65) |
| Moldova | 0.25 (0.19~0.32) | 0.08 (0.05~0.1) | -68 | 1.1 (0.82~1.44) | 0.44 (0.32~0.6) | -2.94 (-3.42~-2.46) |
| Monaco | 0 (0~0) | 0 (0~0) | NA | 0.28 (0.18~0.4) | 0.21 (0.12~0.33) | -1.02 (-1.18~-0.87) |
| Mongolia | 0.37 (0.27~0.49) | 0.28 (0.18~0.39) | -24.32 | 3.37 (2.43~4.39) | 1.48 (0.99~2.08) | -2.94 (-3.45~-2.43) |
| Montenegro | 0.01 (0.01~0.01) | 0.01 (0~0.01) | 0 | 0.26 (0.17~0.37) | 0.2 (0.13~0.3) | -0.85 (-1.07~-0.63) |
| Morocco | 5.94 (4.45~7.81) | 3.34 (2.3~4.95) | -43.77 | 4.61 (3.47~6.03) | 1.72 (1.18~2.55) | -3.69 (-4.11~-3.27) |
| Mozambique | 26.44 (18.95~36.09) | 61.18 (37.51~87.98) | 131.39 | 45.77 (32.86~62.34) | 45.62 (27.92~66.01) | 1.25 (0.72~1.79) |
| Myanmar | 12.18 (8.23~17.8) | 26.09 (16.98~37.81) | 114.2 | 5.51 (3.77~8.07) | 8.7 (5.65~12.59) | 1.49 (1.17~1.82) |
| Namibia | 2.93 (1.68~3.98) | 3.01 (1.66~4.95) | 2.73 | 46.82 (28.01~63.72) | 23.43 (13.09~38.44) | -2.01 (-2.37~-1.66) |
| Nauru | 0.01 (0.01~0.02) | 0.01 (0~0.01) | 0 | 24.94 (16.67~36.87) | 13.39 (8.29~21.43) | -2.37 (-2.73~-2.01) |
| Nepal | 4.38 (3.24~5.73) | 1.57 (1.07~2.14) | -64.16 | 4.51 (3.37~5.94) | 0.84 (0.57~1.15) | -6.09 (-6.6~-5.57) |
| Netherlands | 0.56 (0.45~0.69) | 0.2 (0.14~0.29) | -64.29 | 0.69 (0.55~0.84) | 0.28 (0.19~0.41) | -3.83 (-4.24~-3.42) |
| New Zealand | 0.1 (0.07~0.13) | 0.09 (0.06~0.13) | -10 | 0.53 (0.38~0.72) | 0.45 (0.3~0.65) | -0.97 (-1.34~-0.59) |
| Nicaragua | 0.17 (0.13~0.21) | 0.16 (0.12~0.21) | -5.88 | 0.9 (0.71~1.14) | 0.44 (0.32~0.58) | -1.88 (-2.61~-1.16) |
| Niger | 25.05 (17.94~32.88) | 68.94 (43.6~97.34) | 175.21 | 75.4 (53.96~99.19) | 74.83 (47.56~105.25) | -0.31 (-0.55~-0.07) |
| Nigeria | 246.65 (153.82~383.45) | 420.1 (255.55~689.04) | 70.32 | 62.21 (39.15~97.01) | 39.48 (23.94~64.84) | -1.76 (-2.3~-1.22) |
| Niue | 0 (0~0) | 0 (0~0) | NA | 10.58 (6.74~15.91) | 4.91 (2.58~7.86) | -3.23 (-3.42~-3.04) |
| North Korea | 15.6 (9.51~23.95) | 2.11 (1.31~3.23) | -86.47 | 13.7 (8.42~21.05) | 1.63 (1.01~2.48) | -7.77 (-8.04~-7.49) |
| North Macedonia | 0.1 (0.08~0.13) | 0.03 (0.02~0.04) | -70 | 0.99 (0.76~1.25) | 0.29 (0.21~0.4) | -4.97 (-5.39~-4.55) |
| Northern Mariana Islands | 0.03 (0.02~0.04) | 0.02 (0.01~0.02) | -33.33 | 8.47 (5.6~12.13) | 8.1 (5.46~11.62) | -0.16 (-0.83~0.51) |
| Norway | 0.11 (0.09~0.14) | 0.04 (0.03~0.07) | -63.64 | 0.53 (0.42~0.66) | 0.18 (0.11~0.3) | -3.5 (-4.37~-2.63) |
| Oman | 0.23 (0.16~0.32) | 0.13 (0.09~0.17) | -43.48 | 3.42 (2.39~4.8) | 0.61 (0.45~0.81) | -6.07 (-6.37~-5.76) |
| Pakistan | 118.9 (91.47~151.95) | 275.77 (194.25~373.82) | 131.93 | 24.7 (19.17~31.25) | 23.65 (16.49~32.05) | -0.32 (-0.74~0.11) |
| Palau | 0 (0~0) | 0 (0~0) | NA | 2.29 (1.5~3.37) | 1.3 (0.83~1.96) | -1.86 (-1.93~-1.79) |
| Palestine | 0.31 (0.22~0.44) | 0.17 (0.13~0.22) | -45.16 | 3.53 (2.48~5.04) | 0.67 (0.52~0.86) | -5.24 (-5.6~-4.89) |
| Panama | 0.4 (0.33~0.49) | 0.92 (0.64~1.27) | 130 | 3.12 (2.59~3.77) | 4.42 (3.07~6.15) | 1.8 (1.33~2.27) |
| Papua New Guinea | 2.23 (1.53~3) | 5.17 (3.75~7.25) | 131.84 | 11.52 (8.01~15.34) | 10.19 (7.37~14.32) | -0.52 (-0.81~-0.22) |
| Paraguay | 0.15 (0.12~0.19) | 0.78 (0.52~1.13) | 420 | 0.79 (0.63~0.97) | 2.04 (1.39~2.97) | 3.08 (1.52~4.67) |
| Peru | 3.87 (3.08~4.91) | 6.82 (4.61~10.01) | 76.23 | 3.43 (2.75~4.33) | 3.81 (2.57~5.58) | 0.06 (-1.04~1.17) |
| Philippines | 36.88 (29.66~45.33) | 39.65 (27.84~56.26) | 7.51 | 11.42 (9.13~14.02) | 6.77 (4.75~9.55) | -1.47 (-1.61~-1.34) |
| Poland | 3.08 (2.58~3.66) | 0.22 (0.16~0.29) | -92.86 | 1.6 (1.34~1.88) | 0.12 (0.09~0.16) | -9.4 (-10.64~-8.14) |
| Portugal | 0.55 (0.45~0.66) | 0.09 (0.06~0.12) | -83.64 | 1.1 (0.9~1.33) | 0.2 (0.15~0.26) | -6.88 (-7.82~-5.92) |
| Puerto Rico | 0.29 (0.24~0.35) | 0.08 (0.06~0.1) | -72.41 | 1.53 (1.28~1.85) | 0.46 (0.33~0.62) | -4.69 (-5.64~-3.74) |
| Qatar | 0.05 (0.04~0.07) | 0.05 (0.03~0.07) | 0 | 3.07 (2.16~4.15) | 0.48 (0.34~0.67) | -6.32 (-6.69~-5.95) |
| Republic of Congo | 7.34 (5.16~10.01) | 12.37 (8.1~17.73) | 68.53 | 67.91 (48.64~92.54) | 45.17 (29.41~64.77) | -0.67 (-1.34~-0.01) |
| Romania | 0.49 (0.37~0.64) | 0.39 (0.28~0.5) | -20.41 | 0.44 (0.33~0.58) | 0.5 (0.37~0.66) | 0.42 (-0.24~1.08) |
| Russia | 64.2 (54.53~75.12) | 6.68 (4.94~8.79) | -89.6 | 8.18 (6.95~9.61) | 0.95 (0.71~1.25) | -8.31 (-8.69~-7.93) |
| Rwanda | 10.13 (7.71~13.6) | 8.65 (5.56~12.49) | -14.61 | 35.05 (26.39~47.4) | 13.88 (9.01~20.14) | -3.28 (-3.69~-2.87) |
| Saint Helena | 45.35 (31.86~61.97) | 86.02 (54.87~125.51) | 89.68 | 87.25 (61.19~119.06) | 68.14 (43.42~99.33) | -0.49 (-0.82~-0.15) |
| Saint Kitts and Nevis | 0 (0~0) | 0 (0~0.01) | NA | 1.09 (0.86~1.38) | 1.44 (0.55~2.36) | 1.56 (0.9~2.21) |
| Saint Lucia | 0.01 (0.01~0.01) | 0.01 (0.01~0.01) | 0 | 0.88 (0.72~1.08) | 0.95 (0.72~1.22) | 1.19 (0.71~1.68) |
| Saint Vincent | 0.06 (0.05~0.07) | 0.08 (0.06~0.1) | 33.33 | 10.61 (8.71~12.75) | 14.43 (11.07~18.5) | 1.25 (0.94~1.57) |
| Samoa | 0.05 (0.03~0.07) | 0.02 (0.01~0.04) | -60 | 6.17 (4.01~9.13) | 2.43 (1.15~3.86) | -4.1 (-4.59~-3.62) |
| San Marino | 0 (0~0) | 0 (0~0) | NA | 0.35 (0.24~0.48) | 0.29 (0.19~0.43) | -0.6 (-0.67~-0.53) |
| Sao Tome and Principe | 0.2 (0.09~0.3) | 0.18 (0.12~0.25) | -10 | 42.59 (21.81~62.57) | 17.24 (11.7~24.49) | -4.05 (-4.8~-3.3) |
| Saudi Arabia | 1.91 (1.3~2.69) | 2.6 (1.77~3.79) | 36.13 | 3.09 (2.09~4.36) | 1.23 (0.84~1.76) | -2.35 (-2.75~-1.96) |
| Senegal | 31.16 (23.09~41.16) | 61.79 (40.87~84.79) | 98.3 | 94.79 (70.03~124.46) | 85.23 (56.74~117.63) | 0.24 (-0.03~0.5) |
| Serbia | 0.21 (0.15~0.27) | 0.08 (0.05~0.11) | -61.9 | 0.47 (0.35~0.63) | 0.21 (0.15~0.3) | -2.78 (-3.14~-2.41) |
| Seychelles | 0.03 (0.02~0.04) | 0.02 (0.01~0.02) | -33.33 | 7.12 (5.49~9.03) | 3.42 (2.54~4.55) | -1.82 (-2.27~-1.36) |
| Sierra Leone | 12.13 (8.3~16.79) | 35.84 (23.9~48.92) | 195.47 | 71.2 (48.64~97.94) | 84.21 (57.38~115.34) | 1.17 (0.82~1.53) |
| Singapore | 0.24 (0.19~0.3) | 0.05 (0.03~0.06) | -79.17 | 1.22 (0.99~1.49) | 0.14 (0.11~0.18) | -6.77 (-7.79~-5.73) |
| Slovakia | 0.2 (0.15~0.26) | 0.07 (0.05~0.1) | -65 | 0.78 (0.59~1.01) | 0.3 (0.21~0.43) | -2.57 (-3.54~-1.59) |
| Slovenia | 0.14 (0.1~0.2) | 0.02 (0.01~0.03) | -85.71 | 1.41 (0.98~1.99) | 0.24 (0.16~0.33) | -4.22 (-5.65~-2.77) |
| Solomon Islands | 0.53 (0.34~0.8) | 1.17 (0.72~1.7) | 120.75 | 36.18 (23.36~54.55) | 35.4 (22.04~51.37) | -0.11 (-0.36~0.13) |
| Somalia | 17.13 (10.34~25.33) | 46.57 (30.31~67.39) | 171.86 | 60.53 (36.33~91.65) | 57.13 (36.63~83.6) | 0.07 (-0.1~0.24) |
| South Africa | 108.05 (87.42~131.64) | 41.43 (25.05~62.59) | -61.66 | 54.37 (43.67~66.02) | 12.9 (7.89~19.3) | -4.02 (-5.47~-2.54) |
| South Korea | 1.36 (1.1~1.69) | 0.4 (0.31~0.51) | -70.59 | 0.49 (0.4~0.61) | 0.17 (0.13~0.22) | -4.47 (-5.01~-3.93) |
| South Sudan | 7.68 (4.81~11.55) | 11.02 (6.22~18.48) | 43.49 | 34.06 (21.44~50.98) | 27.09 (15.24~45.53) | -0.43 (-0.7~-0.16) |
| Spain | 0.81 (0.64~1.03) | 0.44 (0.29~0.66) | -45.68 | 0.42 (0.33~0.53) | 0.22 (0.14~0.32) | -1.54 (-2~-1.08) |
| Sri Lanka | 17.36 (13.16~22.31) | 12.63 (8.41~18.14) | -27.25 | 17.89 (13.62~22.93) | 11.66 (7.8~16.85) | -1.43 (-1.61~-1.24) |
| Sudan | 4.37 (3.17~5.79) | 4.14 (2.44~6.28) | -5.26 | 4.53 (3.28~6.03) | 1.89 (1.12~2.85) | -2.57 (-2.93~-2.2) |
| Suriname | 0.26 (0.16~0.35) | 0.54 (0.38~0.73) | 107.69 | 12.93 (8~17.15) | 18.79 (13.18~25.41) | 1.58 (1.14~2.03) |
| Sweden | 0.24 (0.15~0.35) | 0.17 (0.1~0.28) | -29.17 | 0.6 (0.39~0.9) | 0.38 (0.22~0.61) | -1.57 (-1.95~-1.19) |
| Switzerland | 0.16 (0.12~0.21) | 0.12 (0.09~0.18) | -25 | 0.42 (0.31~0.56) | 0.3 (0.21~0.44) | -1.9 (-2.49~-1.31) |
| Syrian Arab Republic | 1.43 (1.03~1.94) | 0.29 (0.21~0.41) | -79.72 | 2.59 (1.87~3.47) | 0.39 (0.27~0.55) | -6.6 (-7.05~-6.15) |
| Taiwan | 1.84 (1.5~2.24) | 0.5 (0.36~0.67) | -72.83 | 1.54 (1.27~1.87) | 0.41 (0.29~0.54) | -4.71 (-5.37~-4.03) |
| Tajikistan | 0.94 (0.72~1.19) | 0.44 (0.3~0.63) | -53.19 | 3.55 (2.75~4.44) | 0.83 (0.57~1.18) | -6 (-6.7~-5.3) |
| Tanzania | 90.84 (66.08~120.87) | 136.97 (93.13~190.13) | 50.78 | 87.76 (64.13~118.09) | 52.86 (35.4~73.7) | -0.99 (-1.37~-0.61) |
| Thailand | 4.79 (3.35~6.78) | 4.4 (2.8~6.3) | -8.14 | 1.39 (0.98~1.94) | 1.37 (0.87~1.96) | -0.47 (-1.35~0.43) |
| Timor-Leste | 0.61 (0.36~0.86) | 0.87 (0.2~1.28) | 42.62 | 15.72 (9.5~22.22) | 13.88 (3.1~20.46) | -1.16 (-1.8~-0.51) |
| Togo | 13.4 (9.91~17.77) | 14.15 (9.03~19.88) | 5.6 | 83.81 (62.02~110.24) | 35.12 (22.48~49.28) | -3.15 (-3.62~-2.69) |
| Tokelau | 0 (0~0) | 0 (0~0) | NA | 16.19 (10.2~24.6) | 8.04 (4.73~12.5) | -2.62 (-2.71~-2.53) |
| Tonga | 0.05 (0.04~0.07) | 0.04 (0.03~0.06) | -20 | 13.1 (9.72~17.28) | 8.48 (5.35~12.52) | -1.9 (-2.07~-1.73) |
| Trinidad and Tobago | 0.09 (0.07~0.1) | 0.34 (0.21~0.5) | 277.78 | 1.33 (1.08~1.63) | 4.75 (3.05~7.1) | 5.55 (4.22~6.89) |
| Tunisia | 0.86 (0.65~1.14) | 0.4 (0.26~0.57) | -53.49 | 1.99 (1.51~2.59) | 0.65 (0.42~0.93) | -3.91 (-4.06~-3.75) |
| Turkey | 7.54 (5.71~9.78) | 1.77 (1.32~2.33) | -76.53 | 2.47 (1.88~3.2) | 0.4 (0.3~0.52) | -7.52 (-8.39~-6.65) |
| Turkmenistan | 0.5 (0.4~0.62) | 0.6 (0.42~0.83) | 20 | 2.74 (2.2~3.41) | 2.35 (1.67~3.28) | -0.16 (-0.64~0.32) |
| Tuvalu | 0.01 (0.01~0.01) | 0 (0~0.01) | -100 | 19.78 (13.52~28.17) | 8.14 (4.97~12.21) | -3.31 (-3.44~-3.19) |
| Uganda | 17.19 (10.72~24.91) | 56.91 (36.82~82.9) | 231.06 | 24.77 (15.69~36.19) | 30.6 (20.3~44.46) | 0.8 (0.15~1.45) |
| UK | 3.81 (3.17~4.56) | 1.3 (0.95~1.72) | -65.88 | 1.33 (1.1~1.58) | 0.42 (0.31~0.55) | -4.23 (-4.54~-3.91) |
| Ukraine | 2.18 (1.63~2.82) | 0.92 (0.6~1.3) | -57.8 | 0.9 (0.67~1.19) | 0.47 (0.31~0.66) | -1.91 (-2.58~-1.23) |
| United Arab Emirates | 0.16 (0.11~0.23) | 0.17 (0.11~0.25) | 6.25 | 2.25 (1.55~3.14) | 0.44 (0.28~0.63) | -6.03 (-6.47~-5.58) |
| United States Virgin Islands | 0.01 (0~0.01) | 0 (0~0) | -100 | 1.08 (0.8~1.4) | 0.76 (0.51~1.17) | -0.68 (-1.3~-0.05) |
| Uruguay | 0.18 (0.14~0.24) | 0.1 (0.07~0.13) | -44.44 | 1.22 (0.96~1.57) | 0.58 (0.42~0.81) | -3.7 (-4.27~-3.13) |
| USA | 26.04 (21.75~30.67) | 12.31 (10.08~14.84) | -52.73 | 1.93 (1.61~2.27) | 0.83 (0.68~1) | -2.45 (-2.76~-2.14) |
| Uzbekistan | 1.1 (0.82~1.48) | 1.25 (0.93~1.61) | 13.64 | 1.04 (0.79~1.37) | 0.66 (0.5~0.85) | -0.88 (-1.38~-0.37) |
| Vanuatu | 0.06 (0.04~0.1) | 0.11 (0.06~0.17) | 83.33 | 8.42 (4.91~13.17) | 7.26 (3.72~10.97) | -1.27 (-1.58~-0.96) |
| Venezuela | 0.91 (0.75~1.1) | 6.17 (4.3~8.62) | 578.02 | 0.88 (0.72~1.06) | 4.29 (3~5.99) | 5.8 (4.97~6.64) |
| Vietnam | 3.11 (2.04~4.7) | 1.45 (1.03~1.98) | -53.38 | 0.83 (0.55~1.24) | 0.27 (0.2~0.38) | -3.12 (-3.45~-2.79) |
| Yemen | 3.37 (1.79~5.27) | 9.38 (5.18~14.17) | 178.34 | 6 (3.31~9.2) | 5.8 (3.24~8.8) | -0.12 (-0.57~0.34) |
| Zambia | 18.04 (13.44~23.35) | 20.52 (13.53~28.59) | 13.75 | 58.16 (42.8~76.92) | 24.95 (16.4~35.6) | -3.21 (-3.47~-2.95) |
| Zimbabwe | 30.68 (22.81~39.16) | 43.16 (29.18~59.87) | 40.68 | 67.39 (50.61~86.84) | 54.22 (36.68~75.34) | 1.57 (0.41~2.74) |

DALY, disability adjusted life-year; CI, confidence interval; EAPC, estimated annual percentage change; UI, uncertainty interval.


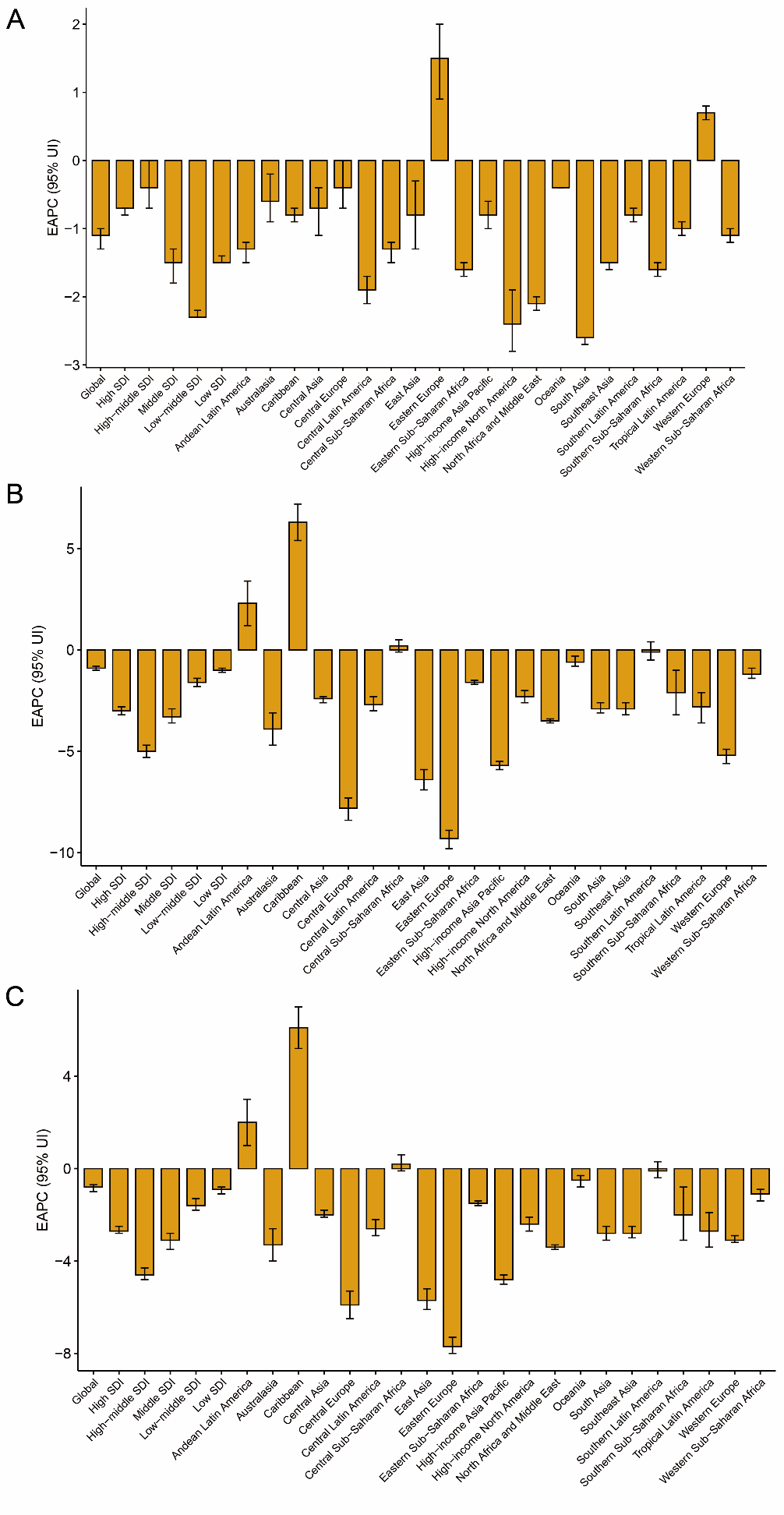


Supplementary Fig. 1 The EAPC of ectopic pregnancy ASR from 1990 to 2019. A. The EAPC of ASIR. B. The EAPC of ASDR. C. The EAPC of age-standardized DALY rate.


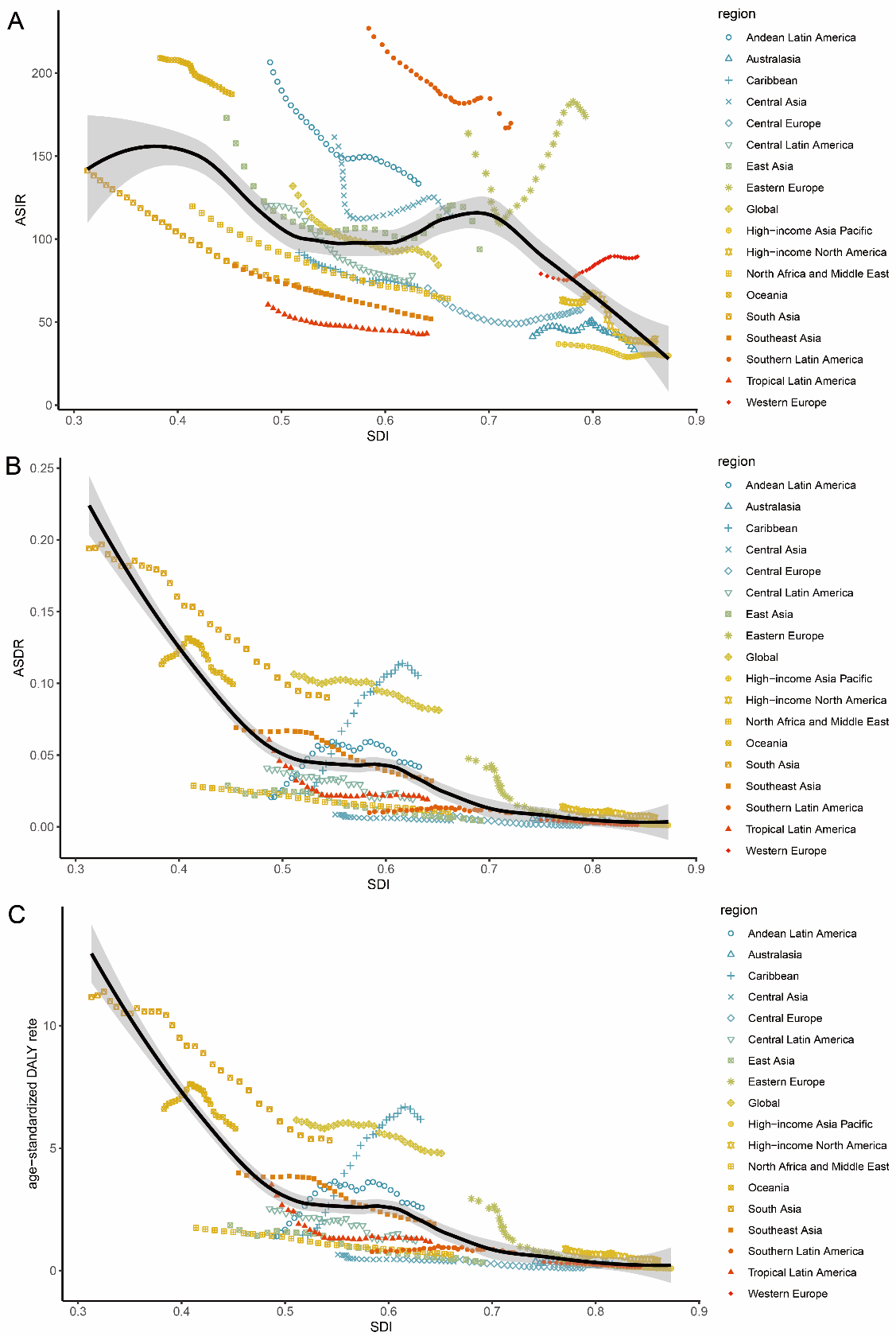


Supplementary Fig. 2 The relationships between age-standardized incidence (A), death (B), and DALY (C) rates of ectopic pregnancy per 100,000 population and SDI among different regions in 2019.


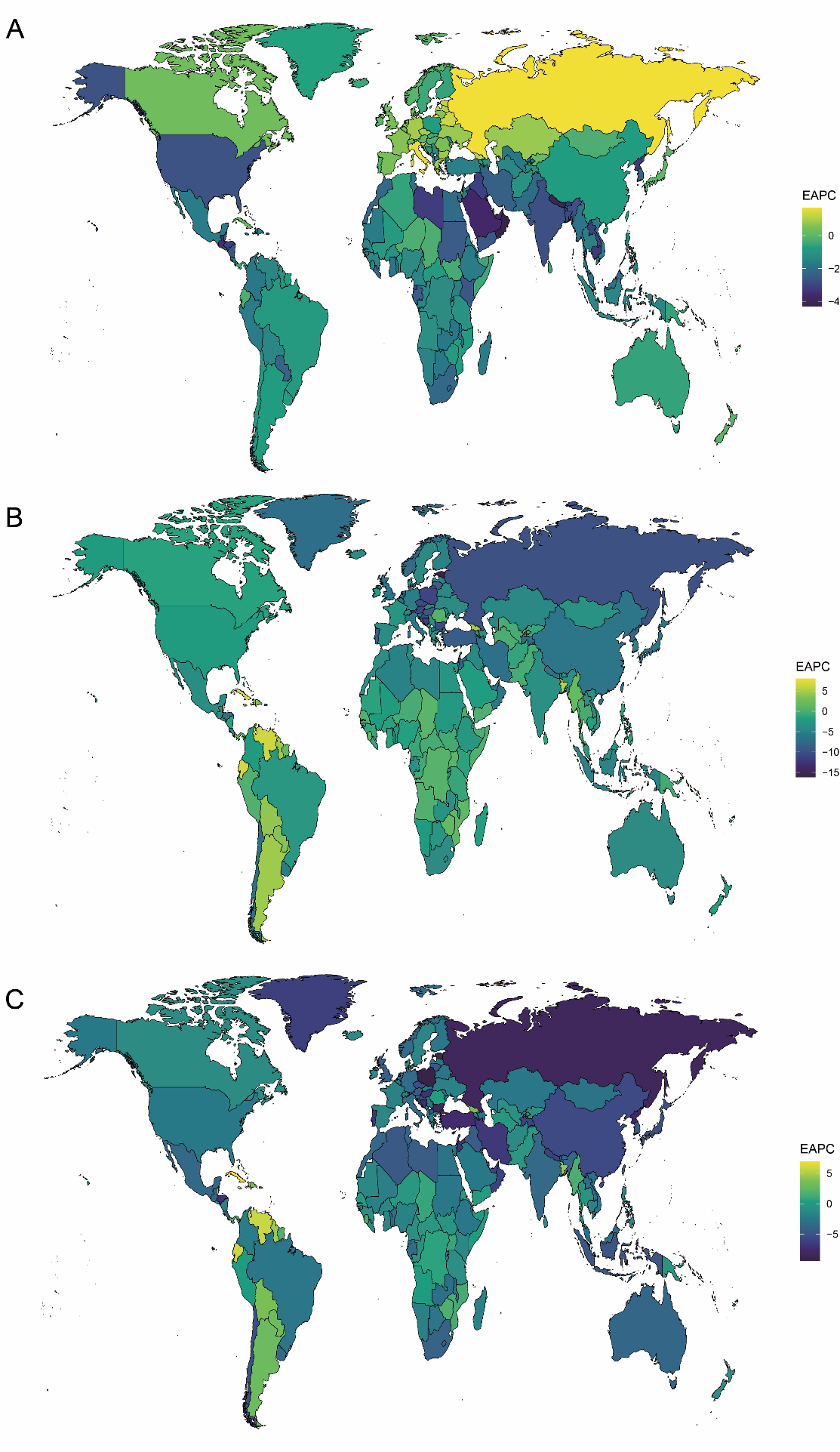


Supplementary Fig. 3 The global EAPC of ectopic pregnancy in 194 countries. A. The EAPC for ASIR. B. The EAPC of ASDR. C. The EAPC of age-standardized DALY rate.
